# Mutagenic activity of quaternary ammonium salt derivatives of carbohydrates

**DOI:** 10.3762/bjoc.12.138

**Published:** 2016-07-12

**Authors:** Barbara Dmochowska, Karol Sikora, Anna Woziwodzka, Jacek Piosik, Beata Podgórska

**Affiliations:** 1 Faculty of Chemistry, University of Gdańsk, Wita Stwosza 63, 80-308 Gdańsk, Poland; 2 Faculty of Pharmacy, Medical University of Gdańsk, Gen. Hallera 107, 80-416 Gdańsk, Poland; 3 Laboratory of Biophysics, Intercollegiate Faculty of Biotechnology, University of Gdańsk and Medical University of Gdańsk, Antoniego Abrahama 58, 80-307 Gdańsk, Poland; 4 Department of Molecular Evolution, University of Gdańsk, Wita Stwosza 59, 80-308 Gdańsk, Poland

**Keywords:** 6-bromohexyl D-glucopyranoside, microbiological mutagenicity test, quaternary ammonium salt

## Abstract

This paper presents a study on a series of quaternary ammonium salt (QAS) derivatives of glucopyranosides with an elongated hydrophobic hydrocarbon chain. The new *N*-[6-(β-D-glucopyranosyloxy)hexyl]ammonium bromides and their *O*-acetyl derivatives were analyzed via ^1^H and ^13^C NMR spectroscopy. The mutagenic activity of the newly synthesized QAS was investigated using two different techniques: The *Vibrio harveyi* luminescence assay and the Ames test. The obtained results support previous findings contesting QAS safety and indicate that QAS, specifically pyridinium derivatives, might be mutagenic.

## Introduction

Carbohydrates and alditols occur broadly in nature and possess many biological functions essential to living organisms. Sugars not only contribute as energetic substances, but also serve as building materials for fungi, microbes, plants, and animals. The polymers (oligosaccharides) of D-glucose found, for example, in wood (cellulose) and D-glucosamine present in shells of crabs and insects (chitin), are the widely known ones [1–2]. Another class of carbohydrate biopolymer derivatives – D-ribose and 2-deoxy-D-ribose – constitutes the backbone of RNA and DNA, respectively.

The mechanisms of molecular recognition and cell interaction are mostly explained by the interaction of carbohydrates with proteins, called lectins, exposed at the cell surface. This process, on the one hand, allows bacteria to interact with other cells during infection, but the same recognition pattern is used to fight bacterial infections. Dendritic cells from mammalian immune system express a variety of sugar-binding proteins (lectins) at their surface. They capture, process, and display antigens to native T-cells and trigger the adaptive immune system [3]. Proteins located at the surface of the cell serve as potential targets for new drugs containing sugars [1–2,4].

Quaternary ammonium salts (QASs) constitute a class of organic compounds with a broad range of applications. A typical QAS consists of a positively charged nitrogen atom with four residues (aliphatic or aromatic). Mostly, one of the aliphatic or aromatic residues possesses hydrophobic properties, whereas the nitrogen group is hydrophilic. The QAS molecules have a typical head/tail structure determining their amphiphilic character [5].

QASs find application in many fields of everyday life, and their usage reaches hundreds of thousands of tons every year – in 2004 that number reached 0.5 million tons [6]. Antibacterial and antifungal action is the significant property of QAS. Therefore, they are being used as disinfectants, starting from hospital services to wood protection and house construction [7–9]. Moreover, QASs are applied in industry, agriculture (as pesticides and herbicides) [10], and chemistry (as catalysts and solvents) [11–12]. QASs are also used as ingredients in hair conditioners, shampoos, and toothpastes [13].

Ionic liquids (IL) are recognized as a particularly interesting group of QASs. These are extensively explored worldwide and find many applications. Because of their unique properties, including low melting point (by definition below 100 °C), high conductivity, high thermal stability, low flammability, and very low (if any) volatility [14], they are called “green chemistry” solvents, and are considered as a good alternative for classical organic solvents. Among many applications, dissolving cellulose (which was impossible for common solvents), depolymerizing nonnatural polymers, and capturing CO_2_ appear particularly interesting [15].

Despite their unique properties, QASs have many drawbacks. Their rapid spread in society and the increasing resistance of pathogenic microorganisms [16] require new chemicals, including QASs, posing new risks for the environment and the living organisms. However, the long-term effects of these new compounds with regard to their possible toxicity toward human cells and aquatic organisms are still unknown [17–18]. Additionally, the impact of these chemicals on the environment is raised; many reports describe accumulation of QASs in sludge, soils, and water [6,10]. It is for this reason that the biodegradation pathways of the newly introduced chemicals need to be thoroughly investigated [19–21]. One of the issues of greatest concern regarding the introduction of newly synthesized chemicals is their possible long-term effect on the living organisms. Many of these chemicals can potentially accumulate in organisms, thus rendering their overall long-term effect difficult to assess. Hence, screening of newly synthesized chemicals for their possible genotoxic activity seems to be a matter of particular importance, as long-term exposure to even low doses of genotoxic compounds can induce mutations, which might lead to cancer [22–24].

To overcome many problems associated with QASs, they were combined with sugars. We intended to obtain biologically active compounds – especially antibacterial and antifungal – with high biocompatibility and good biodegradation properties. Some examples of fused molecules containing QASs and sugar moieties were described before [22,25–26]. It was shown that quaternization of chitosan increases its antimicrobial activity; such polymer is proposed to be used as a wound dressing after surgeries to eliminate infections and to improve the healing process [27–29]. Moreover, the introduction of a sugar moiety to anticancer drug molecules enhanced their activity and selectivity [30–31].

In this paper, we describe process of synthesis, structural characteristics, and mutagenic activity profile of eight new QAS derivatives of 6-aminohexyl D-glucopyranosides. These compounds correspond to previously described derivatives with two carbon atom spacers dividing the sugar moiety and the ammonium group [22]. Here, we synthesized compounds with a long hydrocarbon chain (containing six carbon atoms), which will enable us to analyze the influence of the distance between QASs and the sugar groups on the efficiency of the synthesis process and the influence on their biological activity.

## Results and Discussion

### Chemistry

**Scheme 1:**
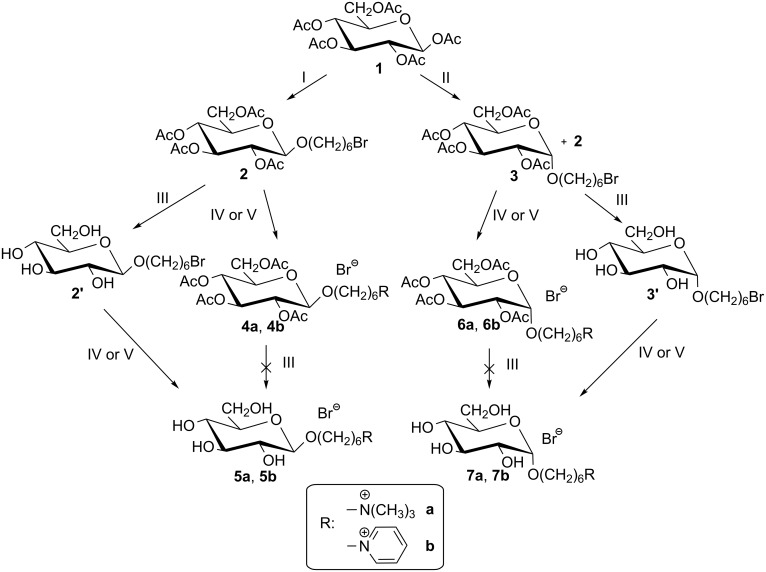
Reagents and conditions; I: HO(CH_2_)_6_Br, BF_3_·Et_2_O/CH_2_Cl_2_, 1 h 0 °C → 3 h, rt, ([32] compound **2** 36% yield); II: HO(CH_2_)_6_Br, BF_3_·Et_2_O/CH_2_Cl_2_, 1 h 0 °C → 72 h (compound **2** 17% yield + compound **3** 24% yield), rt; III: 0.82 M MeONa/MeOH, rt, 24 h; IV: N(CH_3_)_3_/EtOH, 70 °C, 5 h (**4a**, **6a**), 18 h (**5a**, **7a**); V: Py, 70 °C, 24 h (**4b**, **6b**), 36 h (**5b**, **7b**).

We synthesized *N*-[6-(β-D-glucopyranosyloxy)hexyl]ammonium salts to determine the effect of the linker length in QASs on their mutagenic potential (Scheme 1). The main product of the first step of the synthesis was the 1,2-*trans*-glucoside, i.e., 6-bromohexyl 2',3',4',6'-tetra-*O*-acetyl-β-D-glucopyranoside (**2**) in 36% yield [32]. By using a Lewis acid (BF_3_·Et_2_O) as an activator and by extending the reaction time to 72 h, the product with configuration α-D-gluco, i.e., 6-bromohexyl 2',3',4',6'-tetra-*O*-acetyl-α-D-glucopyranoside (**3**) in 24% yield and 6-bromohexyl 2',3',4',6'-tetra-*O*-acetyl-β-D-glucopyranoside (**2**) in 17% yield were obtained. 6-Bromohexyl 2',3',4',6'-tetra-*O*-acetyl-β-D-glucopyranoside (**2**) and 6-bromohexyl 2',3',4',6'-tetra-*O*-acetyl-α-D-glucopyranoside (**3**) were then reacted with trimethylamine in ethanol and with pyridine to assess the effect of the sugar substituent on the course of quaternization. Compounds **4a**, **4b**, **6a**, **6b** were obtained in almost quantitative yields (92–99%). Since de-*O*-acetylated salts could not be obtained using sodium methanolate in methanol, an alternative route involving the reaction of 6-bromohexyl D-glucopyranoside (**2′** [32] or **3′**) with tertiary amines (trimethylamine in ethanol and with pyridine) was applied; the yields were of 90–94%. Shifting the pyranose leaving group (halogen) by six carbon atoms from C1 makes it easier to design the QAS. All the newly synthesized *N*-[6-(β-D-glucopyranosyloxy)hexyl]ammonium bromides were water soluble. The identities of all compounds were confirmed by ^1^H and ^13^C NMR.

### Mutagenic activity of QAS

QASs are generally recognized as safe compounds. Nevertheless, considering our previous observations on the pronounced mutagenic activity of QAS derivatives with two carbon atom spacers dividing the sugar moiety and the ammonium group [22], we investigated the mutagenic activity of newly synthesized QAS with a carbohydrate spacer containing six carbon atoms.

Two different bacterial mutagenicity assays were applied. In the first approach, a recently developed *Vibrio harveyi* bioluminescence assay was used. This assay has been reported as being highly sensitive and therefore capable for antimutagenicity screening [33–34]. Moreover, all QASs synthesized in this work were tested for their mutagenic activity using the *Salmonella typhimurium* TA98 strain in the Ames test. The Ames test is a well-established and routinely used bacterial mutagenicity assay for examining the safety of newly obtained compounds before they are commercially available.

**Figure 1:**
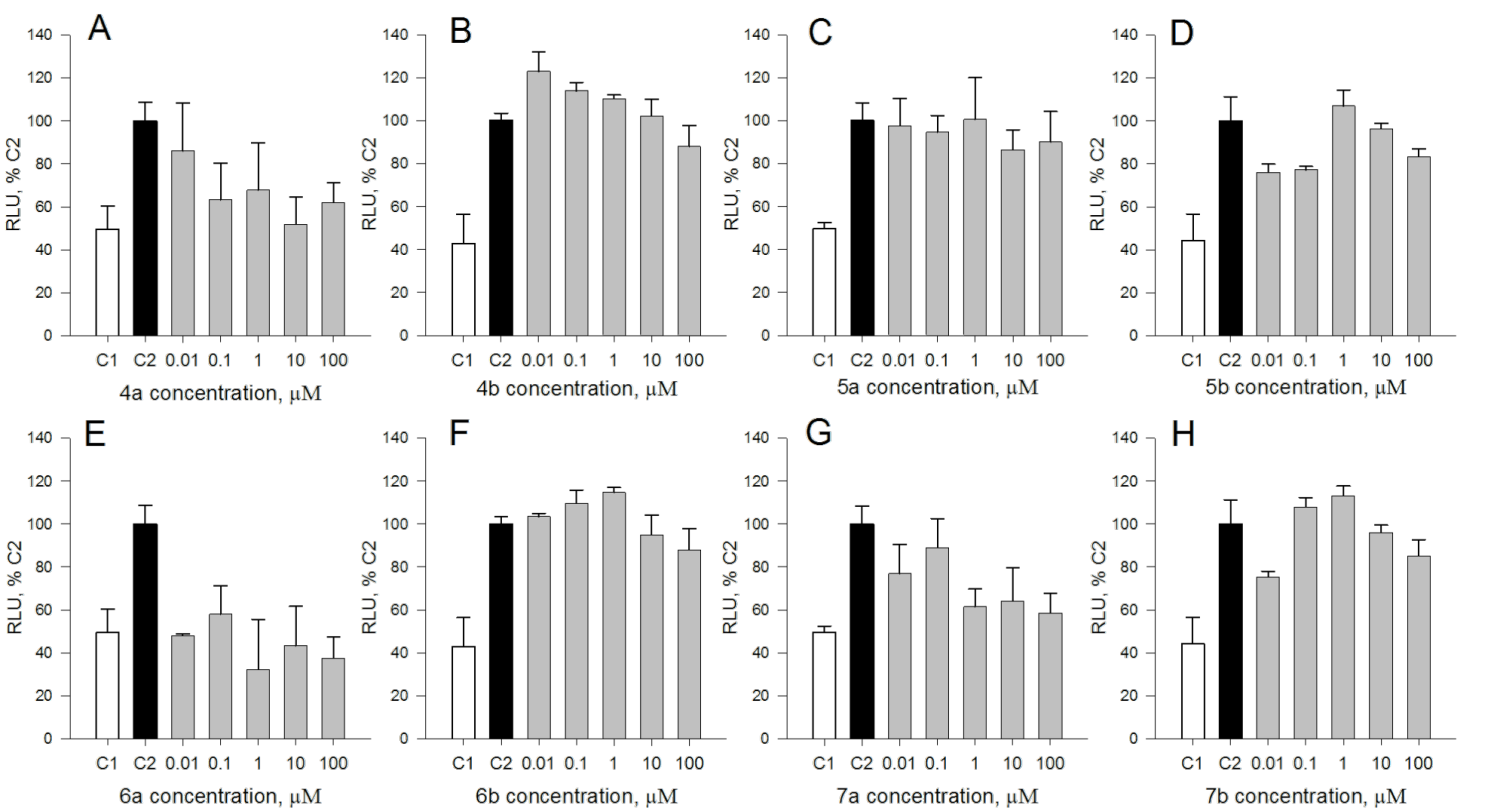
Mutagenic activity of the QASs in the *Vibrio harveyi* A16 strain bioluminescence assay. A, **4a**; B, **4b**; C, **5a**, D, **5b**; E, **6a**; F, **6b**; G, **7a**; H, **7b**. C1 (marked in white), negative (water) control; C2 (marked in black), positive control (6-chloro-9-[3-(2-chloroethylamino)propylamino]-2-methoxyacridine dihydrochloride /ICR191/, 100 nM). Bars indicate mean values (± standard deviation) of bacterial luminescence, expressed as relative light units (RLU) per A_575_ of bacterial culture. Luminescence of the positive control (C2) was considered 100%.

**Figure 2:**
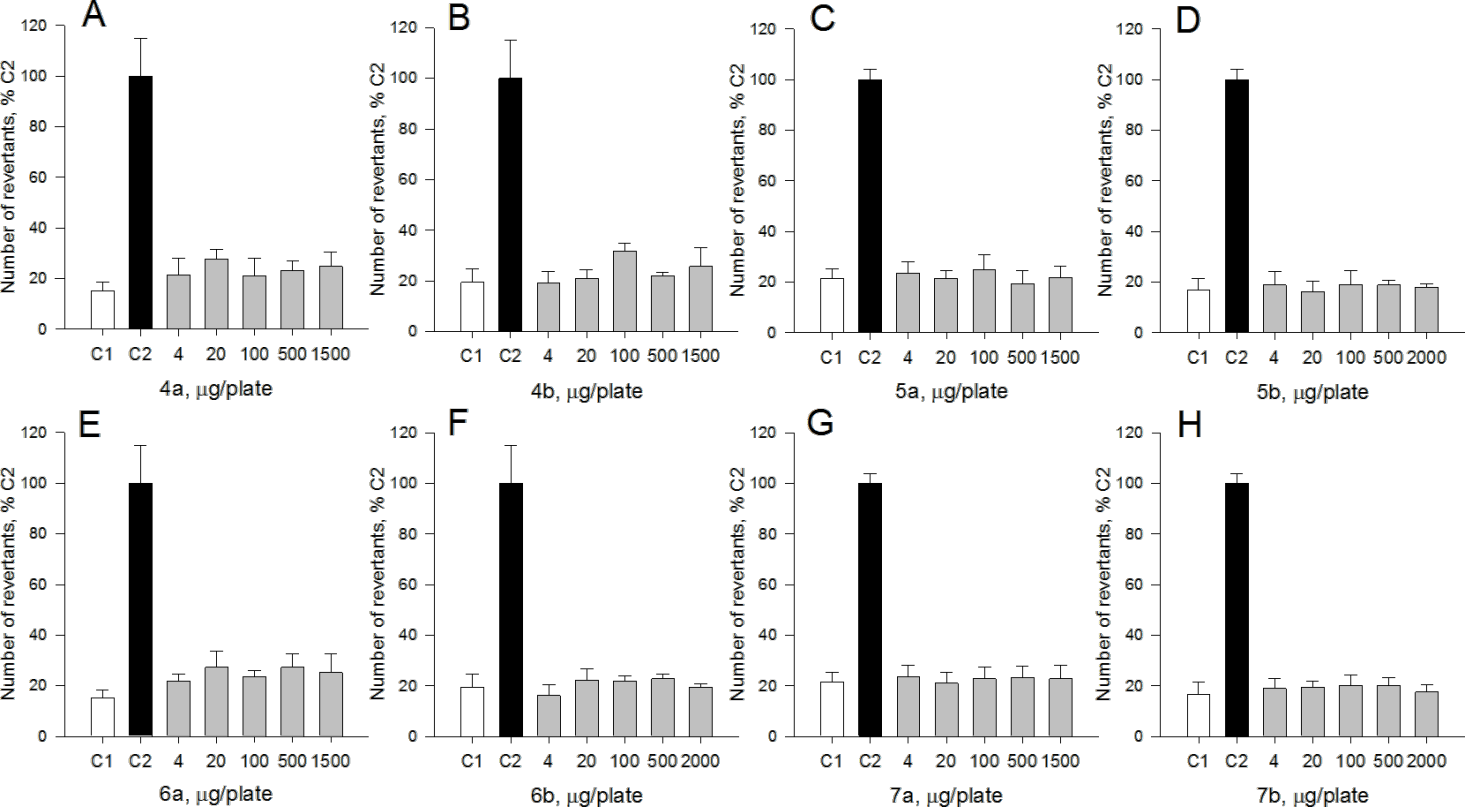
Mutagenic activity of the QASs in the Ames test with histidine-dependent *Salmonella typhimurium* TA98 strain. A, **4a**; B, **4b**; C, **5a**; D, **5b**; E, **6a**; F, **6b**; G, **7a**; H, **7b**. C1 (marked in white), negative (water) control; C2 (marked in black), positive control (2-amino-3-methylimidazo[4,5-*f*]quinoline [IQ], 50 nmol/plate). Results are reported as percentage number of revertants observed for the positive control (C2). Bars indicate mean values from three plates ± standard deviation.

In this work, five out of eight tested QASs, namely, **4b**, **5a**, **5b**, **6b**, and **7b** exhibited a substantial mutagenic activity in the *Vibrio* bioluminescence assay (Figure 1). The extent of their mutagenic activity was comparable to that of a model acridine mutagen, ICR191, used as a positive control. Compounds **4a**, **6a**, and **7a** did not display mutagenic activity in the *Vibrio* luminescence assay at all. Apart from *N*-[6-(β-D-glucopyranosyloxy)hexyl]-*N*,*N*,*N*-trimethylammonium bromide (**5a**), pyridinium salts tend to be more mutagenic than their trimethylammonium counterparts. Moreover, the position of the hexyl chain occurs to be important for mutagenicity of trimethylammonium salts; compounds with an hexyl chain in equatorial position (**4a**, **5a**) are more mutagenic than those with an hexyl chain in axial position (**6a**, **7a**). By contrast, in the corresponding analyses conducted with *Salmonella typhimurium* TA98, no mutagenic effects of the eight tested QASs were observed (Figure 2). Such results are consistent with previously published data indicating that the *Vibrio harveyi* assay can detect very weak mutagenic activity [22,34].

Despite the existing assumption about the general safety of QASs [35–37], our findings suggest that at least some of the QASs might exhibit genotoxic potential. Our observations are in agreement with the previous reports demonstrating the genotoxic potential of QASs. In 2007, Ferk et al. [38] demonstrated genotoxic activity of two commonly used QASs, benzalkonium chloride and dimethyldioctadecylammonium bromide. Although tested compounds were nonmutagenic in bacteria, further analysis on eukaryotic cells revealed their significant genotoxic effects. Moreover, described genotoxicity of QASs toward plant tissues indicates their significance as environmental genotoxins [38]. In the 2011 paper [22], we described a pronounced mutagenic activity of QASs containing a carbohydrate moiety with a two-carbon atom linker in the *Vibrio harveyi* bioluminescence assay. Here, we provide evidence for the mutagenic potential of corresponding QAS–carbohydrate derivatives with a longer, six-carbon atom linker. This suggests that the introduction of a longer hydrocarbon chain does not affect the genotoxic potential of the QASs, indicating the need for other structural modifications that might minimize genotoxic activity of the QAS–carbohydrate derivatives.

## Conclusion

Most of the newly-synthesized QAS were proven to be mutagenic in the *Vibrio harveyi* bioluminescence assay. Obtained results suggest that at least some of QASs can be genotoxic. Moreover, we observed that pyridinium salts tend to be more mutagenic than trimethylammonium derivatives, whereas the position of the hexyl chain seems to be important for the extent of the mutagenic activity of trimethylammonium salts. Regarding extensive usage of QASs, not only in the industry [6–9], but also as constituents of cosmetics and drugs [10,12–13], further research is needed to assess possible genotoxic activity of both newly developed and commercially available compounds as well as to further develop structural modifications that could increase the safety of QASs, particularly the ones intended for medical usage.

## Supporting Information

File 1 General procedures, analytical data and spectra of all new compounds.
