# Supplementary material for: Mutagenic activity of quaternary ammonium salt derivatives of carbohydrates
Source: Beilstein J Org Chem. 2016 Jul 12;12:1434–9. doi: 10.3762/bjoc.12.138 (PMC4979630; doi:10.3762/bjoc.12.138)
Supplement: File 1 — General procedures, analytical data and spectra of all new compounds. [file Beilstein_J_Org_Chem-12-1434-s001.pdf]

Supporting information

for

Mutagenic activity of quaternary ammonium salt derivatives of carbohydrates

Barbara Dmochowska*[1,§], Karol Sikora[1,2], Anna Woziwodzka[3], Jacek Piosik[3] and Beata Podgórska[4]

[1]Faculty of Chemistry, University of Gdańsk, Wita Stwosza 63, 80-308 Gdańsk, Poland, [2]Faculty of Pharmacy, Medical University of Gdańsk, Gen. Hallera 107, 80-416 Gdańsk, Poland, [3]Laboratory of Biophysics, Intercollegiate Faculty of Biotechnology, University of Gdańsk and Medical University of Gdańsk, Antoniego Abrahama 58, 80-307 Gdańsk, Poland and [4]Department of Molecular Evolution, University of Gdańsk, Wita Stwosza 59, 80-308 Gdańsk, Poland

Email: Barbara Dmochowska - basia.dmochowska@ug.edu.pl

*Corresponding author

[§]Tel. +48 58 523 5069; Fax: +48 58 523 5012;

General procedures, analytical data and spectra of all new compounds

Contents

General procedures **S1**

Synthesis and analysis of new chemicals **S3**

Biological assay **S8**

NMR spectra **S10**

MS spectra **S20**

References **S23**

Positive controls for mutagenicity assays – 2-amino-3-methylimidazo[4,5-*f*]quinoline (IQ, CAS 76180-96-6), a mutagen/carcinogen belonging to the heterocyclic aromatic amines, and 6-chloro-9-[3-(2-chloroethylamino)propylamino]-2-methoxyacridine dihydrochloride (ICR191, CAS 17070-45-0), acridine mutagen – were bought from Toronto Research Chemicals (Toronto, Canada) and Sigma-Aldrich, respectively. Stock solutions of tested compounds were prepared by dissolving their weight amounts in distilled water (concentrations in mM range) and stored in darkness at 4 °C. L-Histidine and D-biotin for the Ames test were purchased from Sigma-Aldrich (St. Louis, USA). Commercial D-glucose (Fluka) and 6-bromohexanol (Sigma-Aldrich) were used.

All reactions were monitored by thin-layer chromatography (TLC) on Kieselgel 60 F_{254} silica gel plates (Merck, 0.20 mm thickness). The spots were detected by spraying with 5% ethanolic H_2SO_4 and charring. ^{1}H NMR and ^{13}C NMR spectra were recorded at 25 °C with a Varian Mercury spectrometer at 400 and 100 MHz, respectively, with Me_4Si as the internal standard. Assignments were made on the basis of homonuclear decoupling experiments, and homo- and heteronuclear correlation. Optical rotations were measured with a Perkin Elmer 343 polarimeter. Mass spectrometry was done using a Bruker Biflex III MALDI–TOF mass spectrometer (with 2,5-dihydroxybenzoic acid (DHB) and α-cyano-4-hydroxycinnamic acid (CHCA) matrix) and a Bruker HCTultra spectrometer with electrospray ionization. High-resolution mass spectrometry (HRMS) data were acquired with an Agilent 6550 (Q-TOF) mass spectrometer using a Zorbax Extended C18 column (2.1 x 50 mm; 1.8 μm) at 25 °C; mobile phase employed water:acetonitrile (95:5) at a flow rate of 0.400 mL/min.

Synthesis and analysis of new chemicals

6-Bromohexyl 2',3',4',6'-tetra-*O*-acetyl-D-glucopyranoside. to a solution of **1** (2 g, 5.12 mmol) and 6-bromohexanol (0.86 mL, 6.31 mmol) in dry CH_2Cl_2 (20 mL) boron trifluoride etherate (3.64 mL, 29.48 mmol) was added. The reaction mixture was stirred for 1 h in ice and 72 h at room temperature (rt); CH_2Cl_2 (4 mL) was added, and the solution was neutralized with saturated aqueous $NaHCO_3$ solution before being washed twice with water. The organic phase was dried over anhydrous magnesium sulfate, filtered, and the filtrate was evaporated in a vacuum. Compounds **2** [S1] (220 mg, 17%) and **3** (310 mg, 24%) were isolated by column chromatography: ethyl acetate-toluene (2:1).

6-Bromohexyl 2',3',4',6'-tetra-*O*-acetyl-α-D-glucopyranoside (3). R_f = 0.54 (ethyl acetate-toluene 1:2), $[\alpha]_D^{20}$ 93.0° (*c* 0.5, $CHCl_3$); ^{1}H-NMR ($CDCl_3$): δ 5.47 (t, 1H, H-3, $J_{3,4}$ 9.6 Hz); 5.06 (d, 1H, H-1, $J_{1,2}$ 4.0 Hz); 5.04 (t, 1H, H-4, $J_{3,4}$ 9.6 Hz); 4.85 (dd, 1H, H-2, $J_{2,3}$ 10.4 Hz); 4.25 (dd, 1H, H-6', $J_{5,6'}$ 4.4 Hz); 4.09 (dd, 1H, H-6, $J_{5,6}$ 2.4 Hz $J_{6',6}$ 12.0 Hz); 4.00 (m, 1H, H-5); 3.69 (dt, 1H, OCH_{2a}); 3.46-3.36 (m, 3H, OCH_{2b}, CH_2Br); 2.09-2.01 (12H, 4 × OAc); 1.87 (m, 2H, CH_2); 1.62 (m, 2H, CH_2); 1.47 (m, 2H, CH_2); 1.39 (m, 2H, CH_2), ^{13}C NMR ($CDCl_3$): 170.81-169.80 (4C, 4 × OO*C*CH_3), 95.92 (C-1), 71.17 (C-2), 70.47 (C-3), 68.91 (C-4), 68.71 (O-CH_2), 67.43 (C-5), 62.21 (C-6), 33.84 (CH_2Br), 32.85; 29.29; 28.04; 25.48 (4C, 4 x CH_2), 20.92–20.82 (4C, 4 x COO*C*H_3); MALDI-TOF MS (DHB): m/z 533.2 and 535.2 ($[M+Na]^+$), 550.4 and 551.5 ($[M+K]^+$).

***N*-[6-(2',3',4',6'-Tetra-*O*-acetyl-β-D-glucopyranosyloxy)hexyl]-*N*,*N*,*N*-trimethylaminium bromide (4a).** 6-Bromohexyl 2',3',4',6'-tetra-*O*-acetyl-β-D-glucopyranoside (**2**) (101.8 mg, 0.20 mmol) and a 33% ethanolic solution of trimethylamine (0.4 mL) were heated in a glass, screw-capped ampoule for 5 h at 70 °C. Analogous as described in [S2]. The yield of compound **4a** was 105.6 mg (93%), R_f = 0.0 (ethyl acetate-toluene 1:2), $[\alpha]_D^{20}$ -14.7° (*c* 0.4,

H_2O); ^{1}H-NMR (D_2O): δ 5.29 (t, 1H, H-3, $J_{3,4}$ 9.6 Hz); 5.05 (t, 1H, H-4, $J_{3,4}$ 9.6 Hz); 4.88 (dd, 1H, H-2, $J_{2,3}$ 9.6 Hz); 4.78 (d, 1H, H-1, $J_{1,2}$ 8.0 Hz); 4.32 (dd, 1H, H-6', $J_{5,6'}$ 4.0 Hz); 4.16 (dd, 1H, H-6, $J_{6',6}$ 12.4 Hz); 3.99 (m, 1H, H-5); 3.85 (dt, 1H, OCH_{2a}); 3.62 (dt, 1H, OCH_{2b}); 3.24 (m, 2H, CH_2-N, *J* 4.4); 3.04 (s, 9H, 3 × CH_3 of $N(CH_3)_3$); 2.07-2.00 (12H, 4 × OAc); 1.73 (m, 2H, CH_2); 1.54 (m, 2H, CH_2); 1.32 (m, 4H, 2 x CH_2), ^{13}C NMR (D_2O): 173.96-172.89 (4C, 4 × OO*C*CH_3), 100.41 (C-1), 73.32 (C-3), 71.92 (C-2), 71.33 (C-5), 70.94 (O-CH_2), 68.60 (C-4), 66.86 (CH_2-N), 62.10 (C-6), 53.03 (3C, $N(CH_3)_3$), 28.56; 25.34; 24.82; 22.42 (4C, 4 x CH_2), 20.38–20.28 (4C, COO*C*H_3); HRMS (ESI): m/z ($[M\text{-}Br]^+$) calcd: 490.2647; found: 490.2643.

***N*-[6-(2',3',4',6'-Tetra-*O*-acetyl-β-D-glucopyranosyloxy)hexyl]pyridinium bromide (4b).** 6-Bromohexyl 2',3',4',6'-tetra-*O*-acetyl-β-D-glucopyranoside (**2**) (100.4 mg, 0.20 mmol) and anhydrous pyridine (0.4 mL) were heated in a screw-capped ampoule at 70 °C for 24 h. Similary as previously described in [S2]. The yield of compound **4a** was 132.4 mg (98% yield), R_f = 0.0 (ethyl acetate-toluene 1:2), $[\alpha]_D^{20}$ -54° (*c* 0.1, H_2O); ^{1}H-NMR (D_2O): δ 8.78-7.99 (m, 5 H, Py); 5.28 (t, 1H, H-3, $J_{3,4}$ 9.6 Hz); 5.03 (t, 1H, H-4, $J_{3,4}$ 9.6 Hz); 4.85 (dd, 1H, H-2, $J_{2,3}$ 9.6 Hz); 4.75 (d, 1H, H-1, $J_{1,2}$ 8.0 Hz); 4.54 (t, 2H, CH_2-N, *J* 7.6); 4.30 (dd, 1H, H-6', $J_{6,6'}$ 12.6 Hz, $J_{5,6'}$ 4.0 Hz); 4.15 (dd, 1H, H-6, $J_{5,6}$ 2.0 Hz), 3.97 (m, 1H, H-5); 3.81 (dt, 1H, OCH_{2a}); 3.58 (dt, 1H, OCH_{2b}); 2.05-1.99 (12H, 4 × OAc); 2.00 (m, 2H, CH_2); 1.50 (m, 2H, CH_2); 1.29 (m, 4H, 2 × CH_2), ^{13}C NMR (H_2O): 173.94-172.88 (4C, 4 × OO*C*CH_3), 146.80-144.39, 128.46 (Py), 100.40 (C-1), 73.29 (C-3), 71.89 (C-2), 71.31 (C-5), 70.93 (O-CH_2), 68.58 (C-4), 62.07 (2C, C-6, CH_2-N), 30.63; 28.54; 25.09; 24.73 (4C, 4 × CH_2), 20.35–20.27 (4C, COO*C*H_3); HRMS (ESI): m/z ($[M\text{-}Br]^+$) calcd: 510.2334; found: 510.2329.

***N*-[6-(2',3',4',6'-Tetra-*O*-acetyl-α-D-glucopyranosyloxy)hexyl]-*N*,*N*,*N*-trimethylammonium bromide (6a).** 6-Bromohexyl 2',3',4',6'-tetra-*O*-acetyl-α-D-glucopyranoside (**3**) (80.0 mg, 0.16 mmol) and a 33% ethanolic solution of trimethylamine

(0.2 mL) were heated in a glass, screw-capped ampoule for 5 h at 70 °C. Analogous as described in [S2]. The yield of compound **6a** was 81.5 mg (92%), R_f = 0.0 (ethyl acetate-toluene 1:2), $[\alpha]_D^{20}$ 93.0^o (*c* 0.2, H_2O); ^{1}H-NMR (D_2O): δ 5.38 (t, 1H, H-3, $J_{3,4}$ 9.6 Hz); 5.12 (d, 1H, H-1, $J_{1,2}$ 4.0 Hz); 5.05 (t, 1H, H-4, $J_{3,4}$ 9.6 Hz); 4.99 (dd, 1H, H-2, $J_{2,3}$ 10.0 Hz); 4.30 (dd, 1H, H-6', $J_{5,6'}$ 4.4 Hz); 4.15 (dd, 1H, H-6, $J_{6',6}$ 12.8 Hz); 4.14 (m, 1H, H-5); 3.70 (dt, 1H, OCH_{2a}); 3.55 (dt, 1H, OCH_{2b}); 3.26 (m, 2H, CH_2-N); 3.05 (s, 9H, 3 × CH_3 of $N(CH_3)_3$); 2.07-2.01 (12H, 4 × OAc); 1.75 (m, 2H, CH_2); 1.61 (m, 2H, CH_2); 1.38 (m, 4H, 2 × CH_2), ^{13}C NMR (D_2O): 173.94-172.82 (4C, 4 × OO*C*CH_3), 95.43 (C-1), 70.89 (C-3), 70.74 (C-4), 68.75 (2C, C-2 and O-CH_2), 67.28 (C-5), 66.87 (CH_2-N), 62.27 (C-6), 53.04 (3C, $N(CH_3)_3$), 28.18; 25.45; 25.18; 22.42 (4C, 4 × CH_2), 20.33–20.27 (4C, COO*C*H_3); HRMS (ESI): m/z ([M-Br]$^+$) calcd: 490.2647; found: 490.2641.

***N*-[6-(2',3',4',6'-Tetra-*O*-acetyl-α-D-glucopyranosyloxy)hexyl]pyridinium bromide (6b).** 6-Bromohexyl 2',3',4',6'-tetra-*O*-acetyl-α-D-glucopyranoside (**3**) (80.0 mg, 0.16 mmol) and anhydrous pyridine (0.4 mL) were heated in a screw-capped ampoule at 70 °C for 24 h. Similary as previously described in [S2]. The yield of compound **6b was** 92.2 mg (99% yield), R_f = 0.0 (ethyl acetate-toluene 1:2), $[\alpha]_D^{20}$ 80 (*c* 0.2, H_2O); ^{1}H-NMR (D_2O): δ 8.79-7.99 (m, 5 H, Py); 5.36 (t, 1H, H-3, $J_{3,4}$ 9.6 Hz); 5.10 (d, 1H, H-1, $J_{1,2}$ 3.6 Hz); 5.04 (t, 1H, H-4, $J_{3,4}$ 9.6 Hz); 4.97 (dd, 1H, H-2, $J_{2,3}$ 10.4 Hz); 4.66 (t, 2H, CH_2-N, *J* 7.6); 4.28 (dd, 1H, H-6', $J_{6,6'}$ 12.8 Hz, $J_{5,6'}$ 4.8 Hz); 4.13 (dd, 1H, H-6, $J_{5,6}$ 2.4 Hz), 4.10 (m, 1H, H-5); 3.67 (dt, 1H, OCH_{2a}); 3.51 (dt, 1H, OCH_{2b}); 2.09-2.00 (12H, 4 × OAc); 1.98 (m, 2H, CH_2); 1.57 (m, 2H, CH_2); 1.34 (m, 4H, 2 x CH_2), ^{13}C NMR (H_2O): 173.93-172.81 (4C, 4 × OO*C*CH_3), 145.83-144.39, 128.47 (Py), 95.42 (C-1), 70.89 (C-3), 70.73 (C-2), 68.74 (2C, C-4 and O-CH_2), 67.26 (C-5), 62.26 (C-6), 62.09 (CH_2-N), 30.63; 28.23; 25.24; 25.08 (4C, 4 x CH_2), 20.31–20.26 (4C, COO*C*H_3); HRMS (ESI): m/z ([M-Br]$^+$) calcd: 510.2334; found: 510.2330.

General procedure of de-*O*-acetylation

To a solution of **2** or **3** or **4a** or **4b** or **6a** or **6b** (0.2 mmol) in MeOH (0.90 mL), MeONa was added (0.3 mL, c = 0.82 M) at room temperature. The mixtures were stirred for 72 h and then neutralized with Dowex 50 WX 8 [H^+]. The solutions were filtered and concentrated under reduced pressure to a thick syrup.

6-Bromohexyl α-D-glucopyranoside (3'). R_f = 0.0 (ethyl acetate-toluene 1:2), $[\alpha]_D^{20}$ 75.0° (c 0.3, MeOH); ^{1}H-NMR (CD_3OD): δ 4.79 (*b*s, 1H, H-1); 3.82 (*b*d, 1H, H-6, $J_{6',6}$ 11.9 Hz); 3.77 (m, 1H, OCH_{2a}); 3.69-3.64 (m, 2H, H-6', H-3, $J_{3,4}$ 9.1 Hz, $J_{5,6'}$ 3.7 Hz); 3.59 (m, 1H, H-5); 3.47 (*b*s, 3H, OCH_{2b}, CH_2Br); 3.40 (*b*d, 1H, H-2, $J_{2,3}$ 9.7 Hz); 3.29 (t, 1H, H-4, $J_{3,4}$ 9.4 Hz); 1.88 (*b*s, 2H, CH_2); 1.67 (*b*s, 2H, CH_2); 1.49 (*b*s, 4H, 2 x CH_2), ^{13}C NMR (CD_3OD): 98.70 (C-1), 73.74 (C-3), 72.27 – 72.20 (C-2, C-5), 70.48 (C-4), 67.52 (O-CH_2), 61.33 (C-6), 32.93 (CH_2Br), 32.56; 29.07; 27.61, 25.41 (4C, 4 × CH_2); MS-ESI: m/z 365.1 and 367.1 ([M+Na]$^+$).

***N*-[6-(β-D-Glucopyranosyloxy)hexyl]-*N,N,N*-trimethylammonium bromide (5a).** 6-Bromohexyl β-D-glucopyranoside (**2'**) [S1] (42.5 mg, 0.12 mmol) and a 33% ethanolic solution of trimethylamine (1 mL) were heated in a glass, screw-capped ampoule for 18 h at 70 °C. Analogous as described in [S2]. The yield of compound **5a** was 36.6 mg (94%); R_f = 0.0 (ethyl acetate-toluene 1:2), $[\alpha]_D^{20}$ -20.3° (c 0.5, H_2O); ^{1}H NMR (D_2O): δ 4.39 (d, 1H, H-1, $J_{1,2}$ 8.0 Hz); 3.87-3.83 (m, 2H, H-6 and O-CH_{2a}, $J_{5,6}$ 2.4 Hz); 3.65 (dd, 1H, H-6', $J_{5,6'}$ 6.0 Hz, $J_{6,6'}$ 12.4 Hz); 3.61 (m, 1H, O-CH_{2b}); 3.42 (t, 1H, H-3, $J_{3,4}$ 9.2 Hz); 3.38 (m, 1H, H-5, $J_{5,6}$ 2.4 Hz); 3.28–3.17 (m, 4H, CH_2-N, H-4, H-2); 3.04 (s, 9H, 3 × CH_3 of $N(CH_3)_3$); 1.73 (m, 2H, CH_2); 1.58 (m, 2H, CH_2); 1.36 (m, 4H, 2 × CH_2), ^{13}C NMR (D_2O): 102.42 (C-1), 76.16; 76.11 (2C: C-3, C-5), 73.41 (2C, C-2 and C-4), 70.54 (O-CH_2), 66.90 (CH_2-N), 61.05 (C-6), 53.06 (3C, $N(CH_3)_3$); 28.67; 25.39; 24.81; 22.42 (4C, 4 × CH_2), HRMS (ESI): m/z ([M-Br]$^+$) calcd: 322.2224; found: 322.2223.

***N*-[6-(β-D-Glucopyranosyloxy)hexyl]pyridinium bromide (5b).** 6-Bromohexyl β-D-glucopyranoside (**2'**) (42.5 mg, 0.12 mmol) and anhydrous pyridine (1 mL) were heated in a screw-capped ampoule at 70 °C for 36 h. Similary as previously described in [S2]. The yield of compound **5b** was 46.3 mg (92%); R_f = 0.0 (ethyl acetate-toluene 1:2), $[\alpha]_D^{20}$ -14.3 (*c* 1.0, H_2O); ^{1}H NMR (D_2O): δ 8.79-7.99 (m, 5H, Py); 4.56 (t, 2H, CH_2-N, *J* 7.2 Hz); 4.37 (d, 1H, H-1, $J_{1,2}$ 8.0 Hz); 3.85-3.82 (m, 2H, H-6 and O-CH_{2a}, $J_{5,6}$ 2.4 Hz); 3.64 (q, 1H, H-6', $J_{6,6'}$ 12.4 Hz); 3.60-3.58 (m, 1H, O-CH_{2b}); 3.40 (dd, 1H, H-3, $J_{3,4}$ 9.2 Hz); 3.38-3.56 (m, 1H, H-5); 3.31 (dd, 1H, H-4, $J_{4,5}$ 9.6 Hz); 3.18 (t, 1H, H-2, $J_{2,3}$ 9.2 Hz); 1.98 (m, 2H, CH_2); 1.55 (m, 2H, CH_2); 1.34 (m, 4H, 2 x CH_2), ^{13}C NMR (D_2O): 145.77-144.42, 128.46 (5C, Py), 102.39 (C-1), 76.15; 76.09 (2C: C-3 and C-5), 73.40 (C-2), 70.51 (O-CH_2), 69.94 (C-4), 62.09 (CH_2-N), 61.05 (C-6), 30.63; 28.68; 25.16; 24.71 (4C, 4 × CH_2), HRMS (ESI): m/z ($[M-Br]^+$) calcd: 342.1911; found: 342.1909.

***N*-[6-(α-D-Glucopyranosyloxy)hexyl]-*N,N,N*-trimethylammonium bromide (7a).** 6-Bromohexyl α-D-glucopyranoside (**3'**) (28.0 mg, 0.08 mmol) and a 33% ethanolic solution of trimethylamine (1 mL) were heated in a glass, screw-capped ampoule for 18 h at 70 °C. Analogous as described in [S2]. The yield of compound **7a** was 37.4 mg (94%); R_f = 0.0 (ethyl acetate-toluene 1:2), $[\alpha]_D^{20}$ 39.8^o (*c* 0.5, H_2O); ^{1}H NMR (D_2O): δ 4.84 (d, 1H, H-1, $J_{1,2}$ 4.0 Hz); 3.79 (dd, 1H, H-6, $J_{5,6}$ 2.4 Hz); 3.68 (dd, 1H, H-6', $J_{6',6}$ 12.0 Hz); 3.67-3.64 (m, 2H, H-5 and O-CH_{2a}); 3.63 (t, 1H, H-3, $J_{3,4}$ 9.4 Hz); 3.48 (dd, 1H, H-2, $J_{2,3}$ 8.0 Hz); 3.48-3.46 (m, 1H, O-CH_{2b}), 3.33 (dd, 1H, H-4, $J_{4,5}$ 9.6 Hz); 3.27–3.23 (m, 2H, CH_2-N, *J* 3.6 Hz); 3.04 (s, 9H, 3 × CH_3 of $N(CH_3)_3$); 1.73 (m, 2H, CH_2); 1.58 (m, 2H, CH_2); 1.36 (m, 4H, 2 x CH_2), ^{13}C NMR (D_2O): 98.31 (C-1), 73.41 (C-5), 72.05 (C-3), 71.54 (C-2), 69.88 (C-4), 68.23 (O-CH_2), 66.91 (CH_2-N), 60.87 (C-6), 53.09-53.00 (3C, $N(CH_3)_3$), 28.52; 25.45; 25.17; 22.44 (4C, 4 × CH_2); HRMS (ESI): m/z ($[M-Br]^+$) calcd: 322.2224; found: 322.2222.

***N*-[6-(α-D-Glucopyranosyloxy)hexyl]pyridinium bromide (7b).** 6-Bromohexyl α-D-glucopyranoside (**3'**) (28.0 mg, 0.08 mmol) and anhydrous pyridine (1 mL) were heated in a screw-capped ampoule at 70 °C for 36 h. Similary as previously described in [S2]. The yield of compound **7b** was 30.2 mg (90%); R_f = 0.0 (ethyl acetate-toluene 1:2), $[\alpha]_D^{20}$ 52.9º (*c* 1.0, H_2O); ^{1}H NMR (D_2O): δ 8.79–7.99 (m, 5H, Py); 4.82 (d, 1H, H-1, $J_{1,2}$ 3.6 Hz); 4.56 (t, 2H, CH_2-N, *J* 7.4 Hz); 3.76 (dd, 1H, H-6, $J_{5,6}$ 2.4 Hz); 3.67 (q, 1H, H-6', $J_{6,6'}$ 12.0 Hz); 3.64-3.56 (m, 3H, O-CH_{2a}, H-3, H-5); 3.47 (dd, 1H, H-2, $J_{2,3}$ 10.0 Hz); 3.45-3.40 (m, 1H, O-CH_{2b}); 3.33 (t, 1H, H-4, $J_{4,5}$ 9.6 Hz); 1.97 (m, 2H, CH_2); 1.55 (m, 2H, CH_2); 1.35 (m, 4H, 2 × CH_2), ^{13}C NMR (D_2O): 145.77-144.41, 128.45 (5C, Py), 98.29 (C-1), 73.41 (C-5), 72.02 (C-3), 71.52 (C-2), 69.86 (C-4), 68.21 (O-CH_2), 62.11 (CH_2-N), 60.85 (C-6), 30.64; 28.54; 25.23; 25.06 (4C, 4 × CH_2); HRMS (ESI): m/z ($[M-Br]^+$) calcd: 342.1911; found: 342.1909.

Biological assay

Vibrio harveyi bioluminescence mutagenicity test

The assay was performed according to Podgórska and Węgrzyn [S3], with the modification introduced by Woziwodzka et al. [S4]. *Vibrio harveyi* A16 strain (a dim *luxE* mutant) [S3] was used as an indicator strain. Bacteria were grown at 30 °C in liquid BOSS nutrient medium (bacto-peptone, 1%; beef extract, 0.3%; glycerol, 0.011 mol L^{-1}; NaCl, 0.51 mol L^{-1}) to OD_{575} = 0.1, when different concentrations of tested QASs were added, and the cultivation was continued for 3 h. The acridine mutagen ICR191 [2-methoxy-6-chloro-9-(3-(2-chloroethyl)aminopropylamino)acridine×2HCl] (100 nM) was used as a positive control. Absorbance (575 nm) and luminescence measurements were performed on a 2300 EnSpire Multimode Plate Reader (PerkinElmer, USA) using the 96-well plates (ViewPlate-96

microplate, White 96-well Microplate with Clear Bottom, PerkinElmer). The results are presented as relative luminescence (RLU) per absorbance unit [S3].

Ames mutagenicity assay

Histidine-dependent (His^-) *Salmonella typhimurium* TA98 strain was obtained from Xenometrics (Stilwell, USA). The *Salmonella* mutagenicity test [S5] was performed as described by Mortelmans and Zeiger [S6] with minor modifications, previously described by Woziwodzka et al. [S4,S7]. A mixture containing 100 μL of the overnight bacterial culture (corresponding to 1×10^8 colony forming units), 100 μL of 3% NaCl, 100 μL of 1 mM histidine–biotin solution, and 100 μL of a test chemical dilution was spread on the glucose minimal (GM) agar plate. Plates were incubated at 37 °C in darkness and after 48 h His^+ revertant colonies were counted. 2-Amino-3-methylimidazo[4,5-*f*]quinoline (IQ), which was reported previously as being strongly mutagenic toward *S. typhimurium* TA98, was used as a positive control. Sterile distilled water was used instead of tested chemicals as a negative control. All experiments were performed in triplicate. The results were reported as means ± standard deviation (SD) per plate for each tested dose and are presented as percentage of a positive control. Any possible toxic effects toward bacteria were determined by observing the auxotrophic background (background lawn). According to the preliminary studies, no toxic activity of tested QASs (at concentrations up to 2 mg/plate for **5b**, **6b**, **7b**, and 1.5 mg/plate for **4a**, **4b**, **5a**, **6a**, **7a**) toward *S. typhimurium* TA98 was observed.

NMR SPECTRA

1. 6-Bromohexyl 2',3',4',6'-tetra-*O*-acetyl-α-D-glucopyranoside (**3**)

^{1}H NMR (400 MHz, $CDCl_3$):

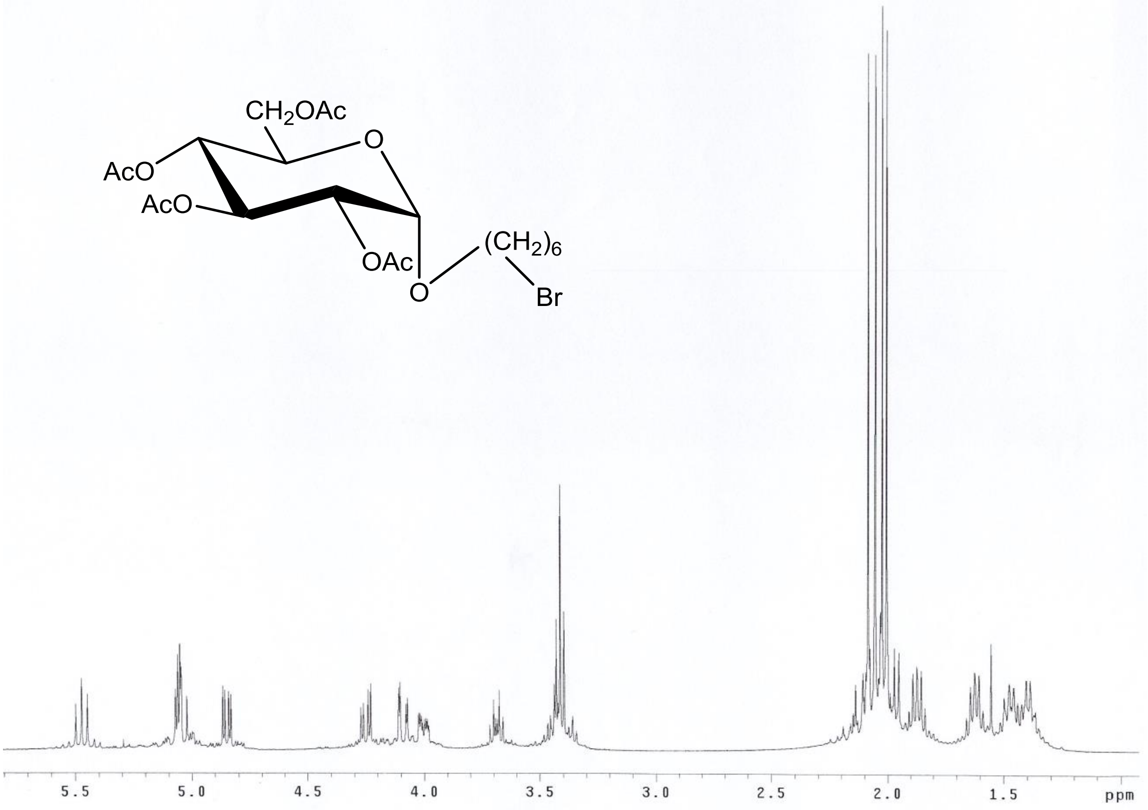

^{13}C NMR (100 MHz, $CDCl_3$):

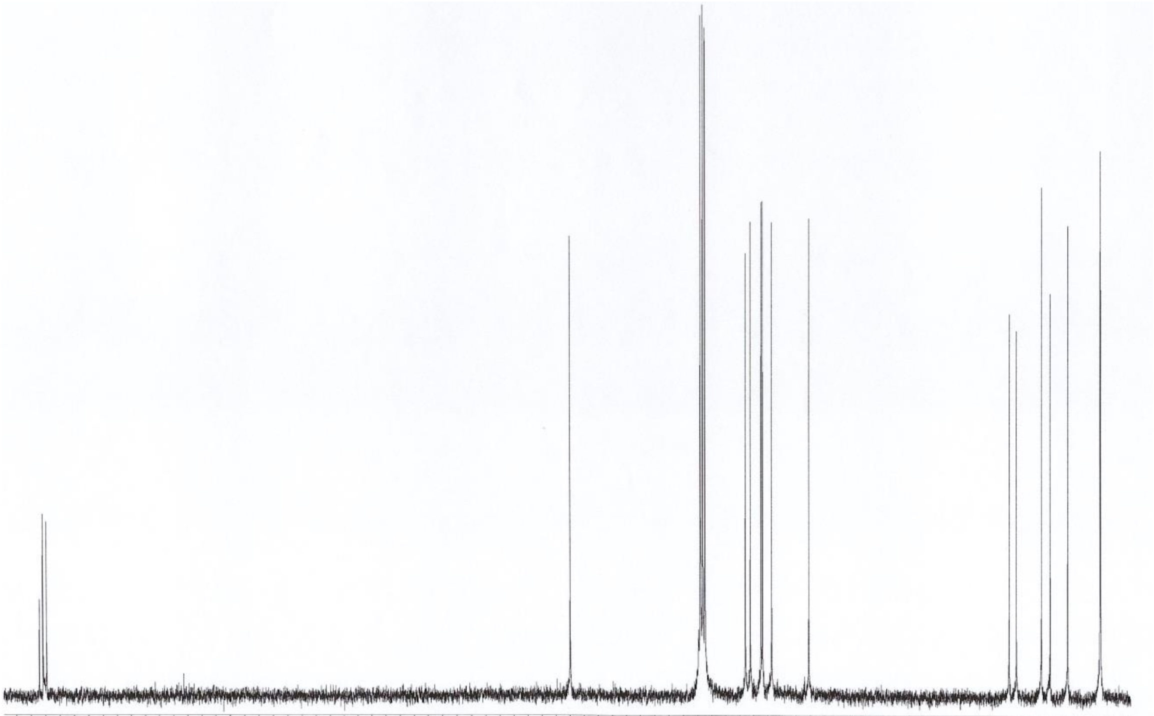

2. 6-Bromohexyl α-D-glucopyranoside (**3’**)

^{1}H NMR (400 MHz, CD_3OD):

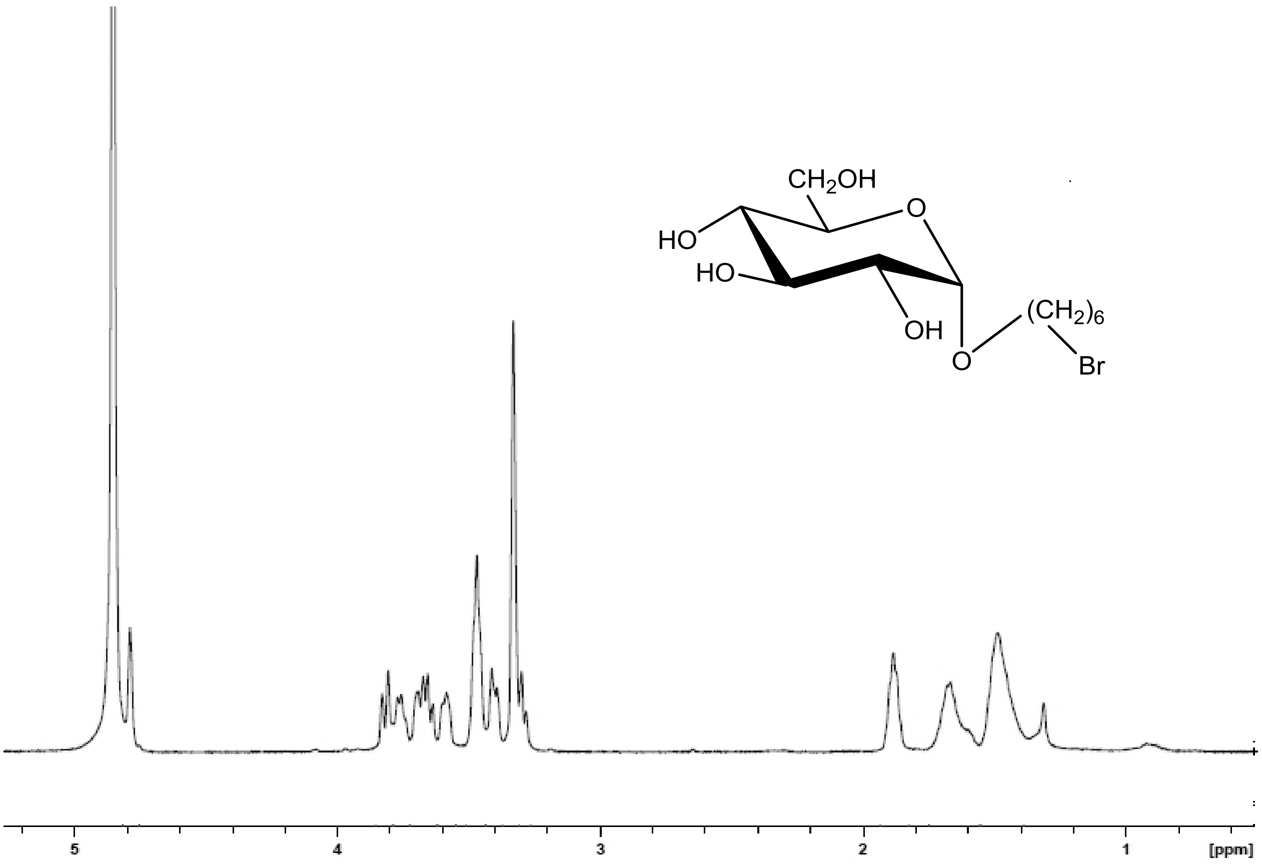

^{13}C NMR (100 MHz, CD_3OD):

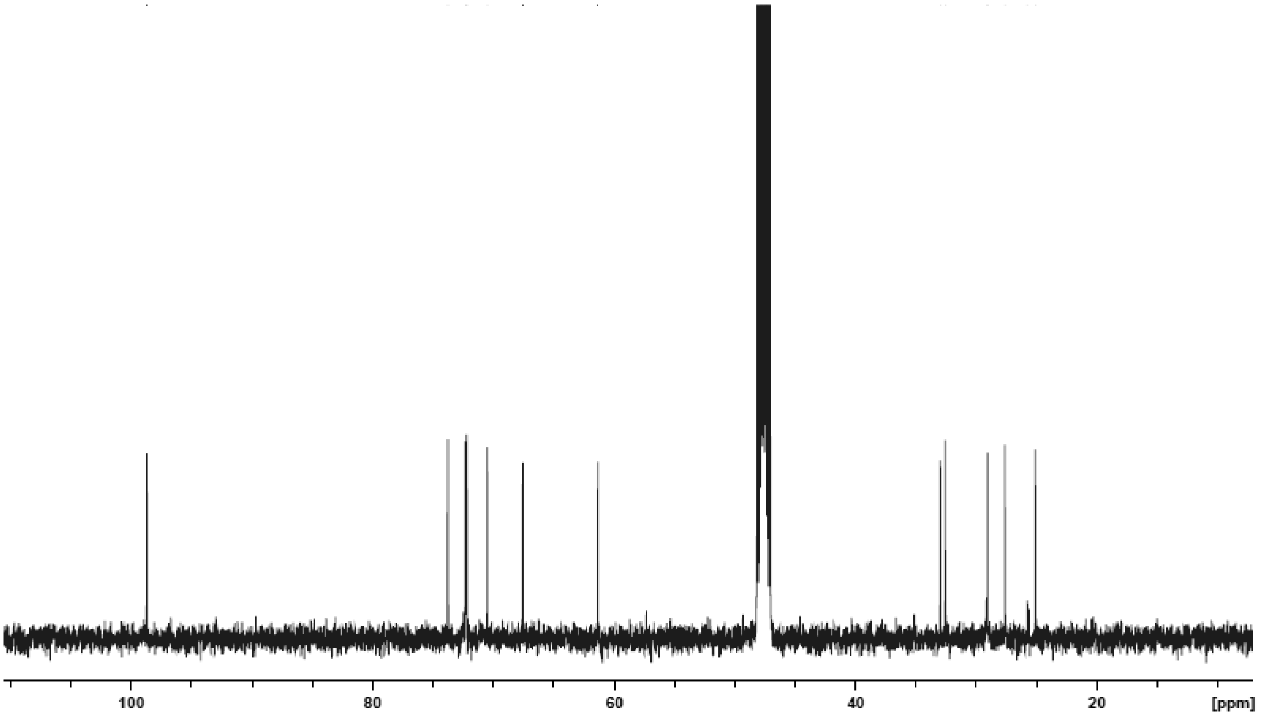

3. *N*-[6-(2',3',4',6'-Tetra-*O*-acetyl-β-D-glucopyranosyloxy)hexyl]-*N*,*N*,*N*-trimethylammonium bromide (**4a**)

^{1}H NMR (400 MHz, D_2O):

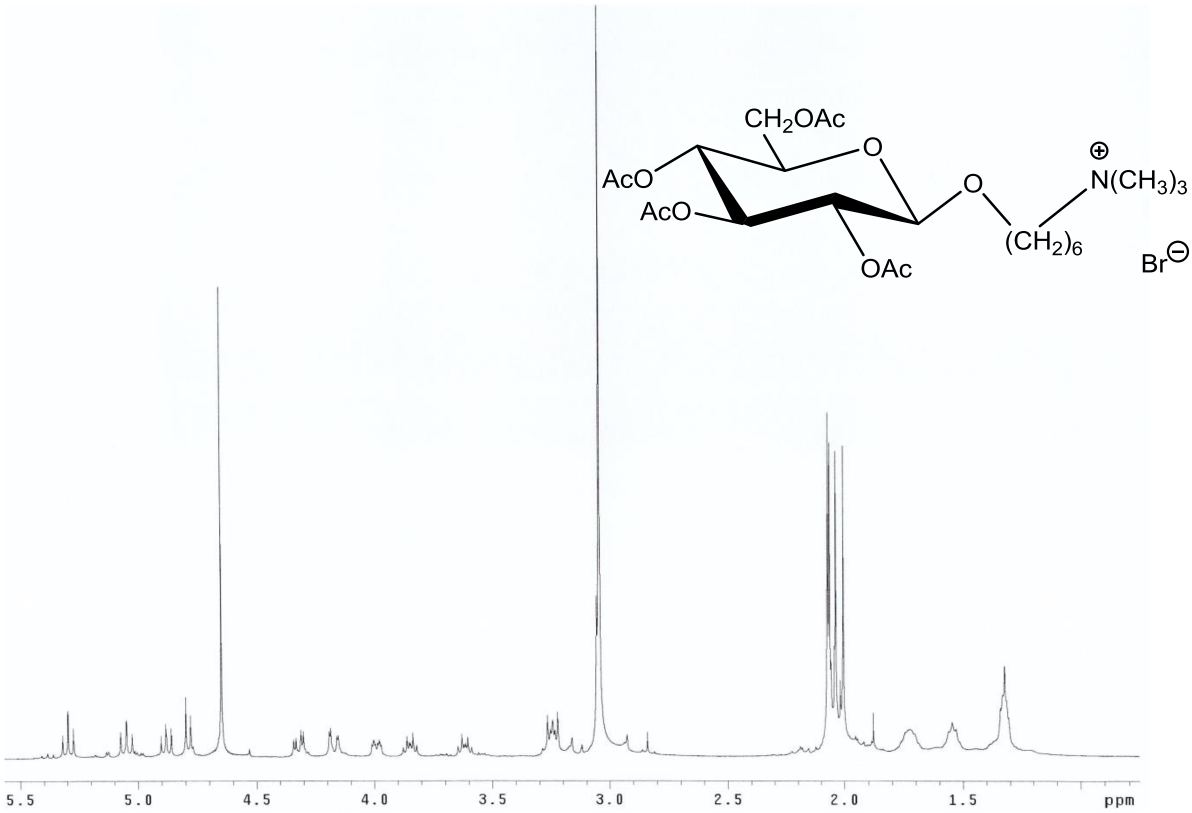

^{13}C NMR (100 MHz, D_2O):

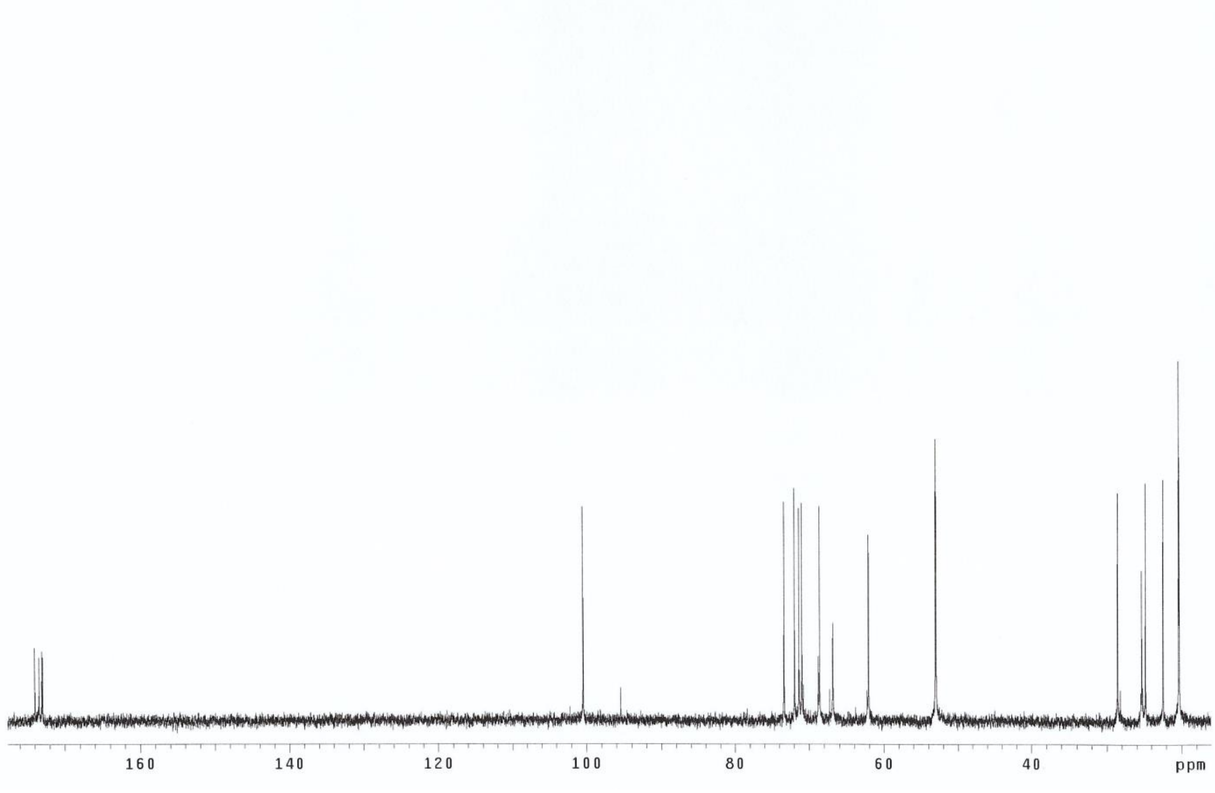

4. *N*-[6-(2',3',4',6'-Tetra-*O*-acetyl-β-D-glucopyranosyloxy)hexyl]pyridinium bromide (**4b**)

^{1}H NMR (400 MHz, D_2O):

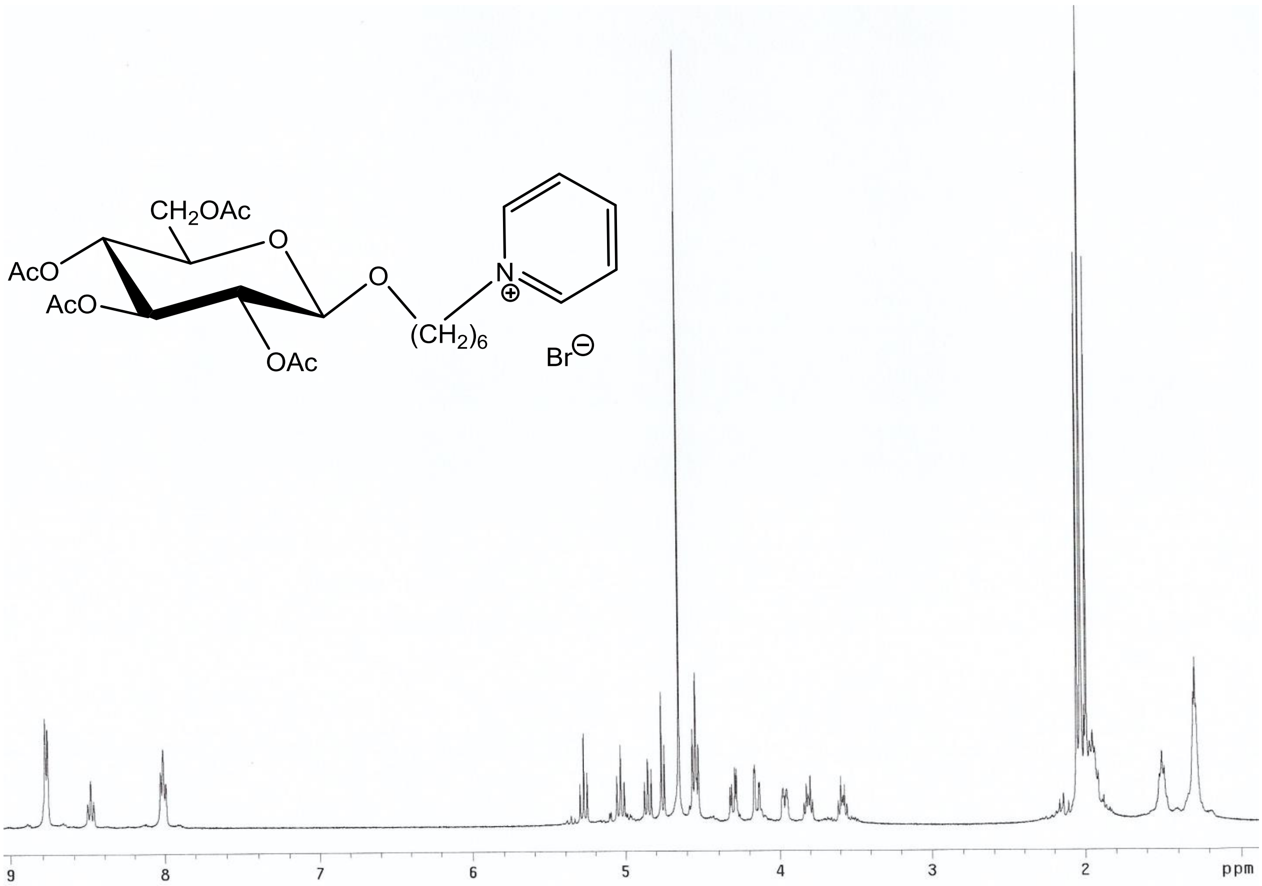

^{13}C NMR (100 MHz, D_2O):

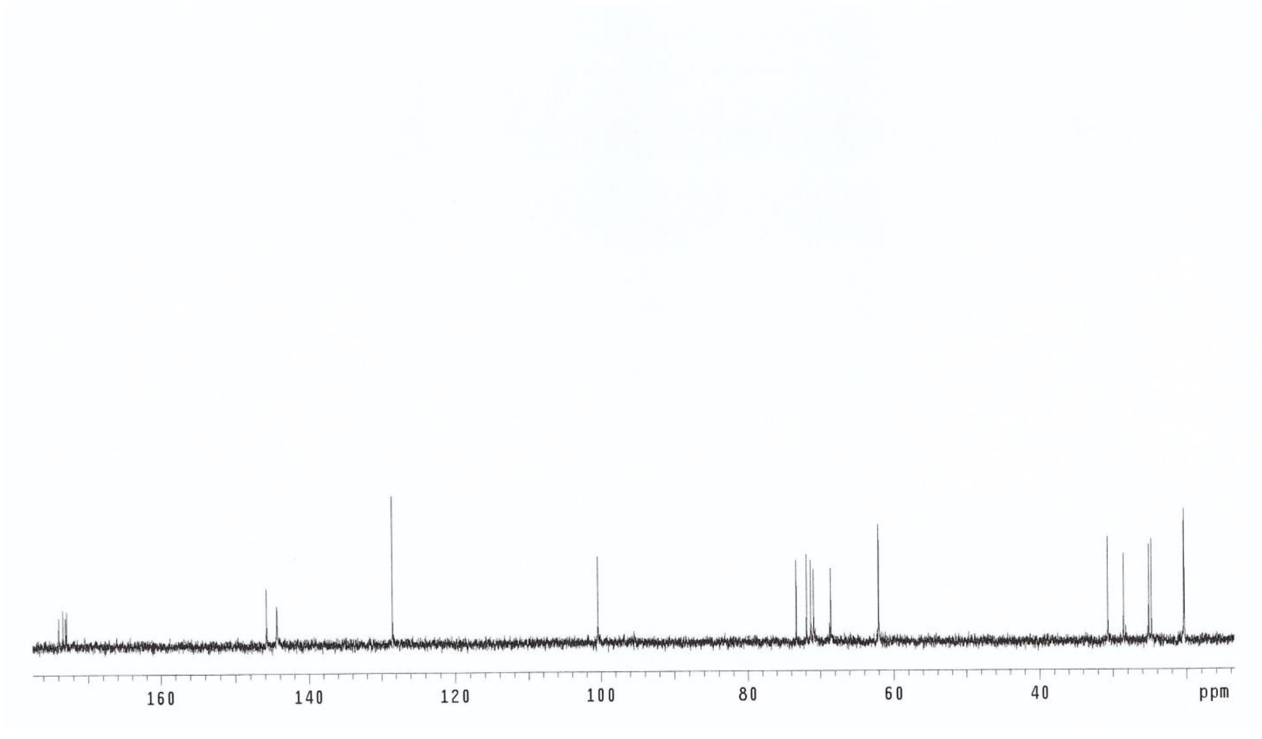

5. *N*-[6-(β-D-glucopyranosyloxy)hexyl]-*N*,*N*,*N*-trimethylammonium bromide (**5a**)

^{1}H NMR (400 MHz, D_2O):

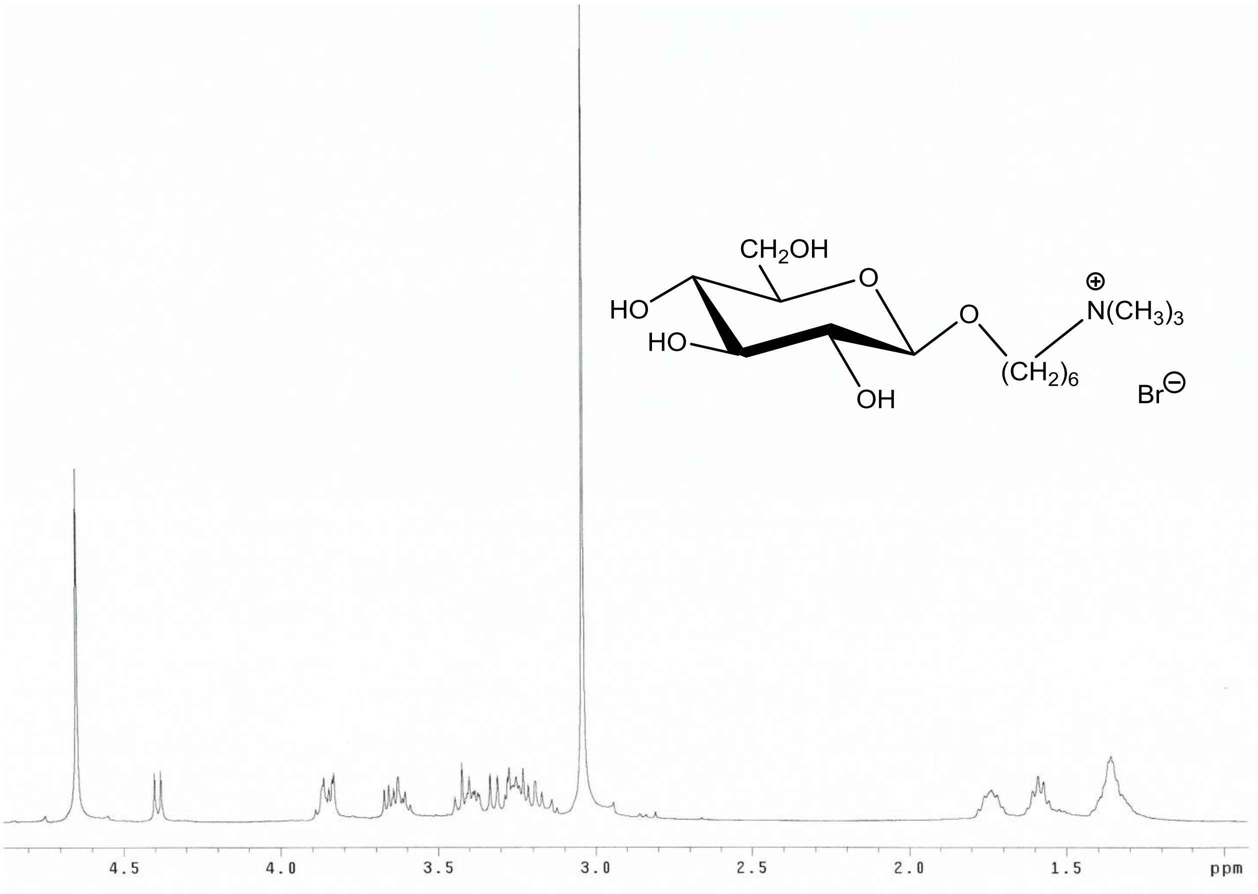

^{13}C NMR (100 MHz, D_2O):

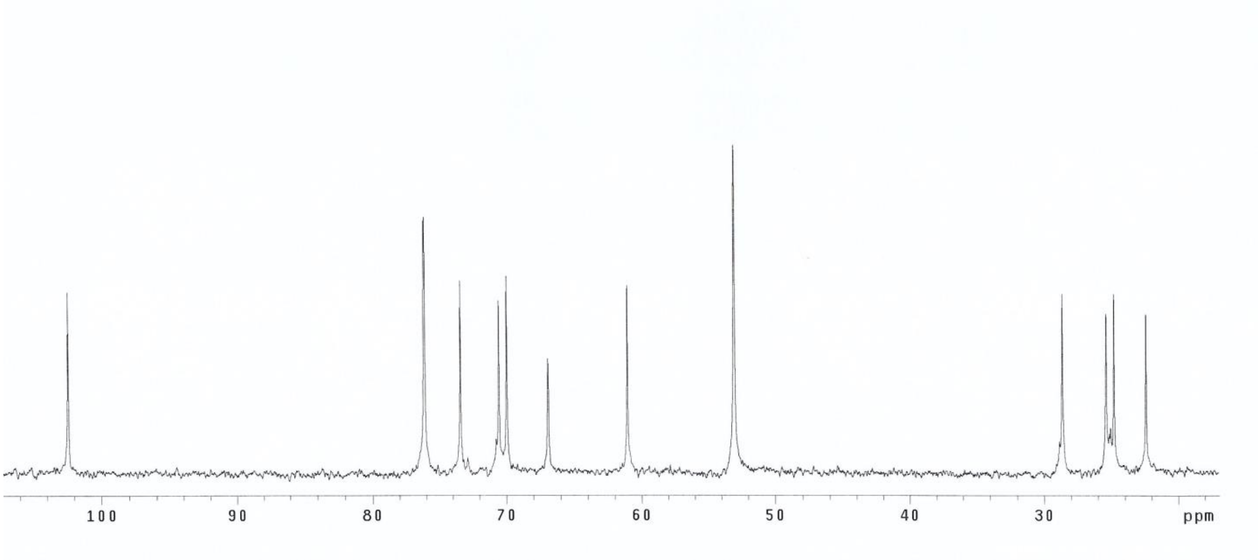

6. *N*-[6-(β-D-glucopyranosyloxy)hexyl]pyridinium bromide (**5b**)

^{1}H NMR (400 MHz, D_2O):

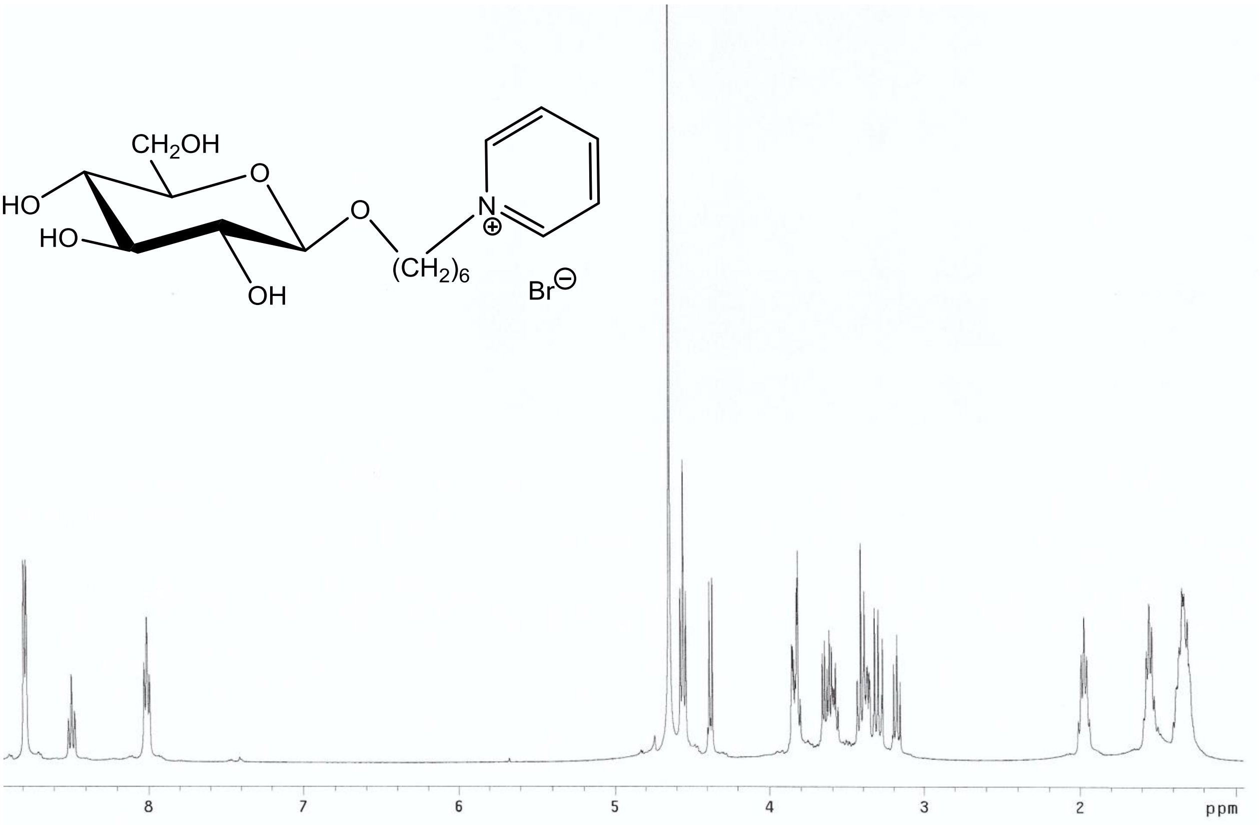

^{13}C NMR (100 MHz, D_2O):

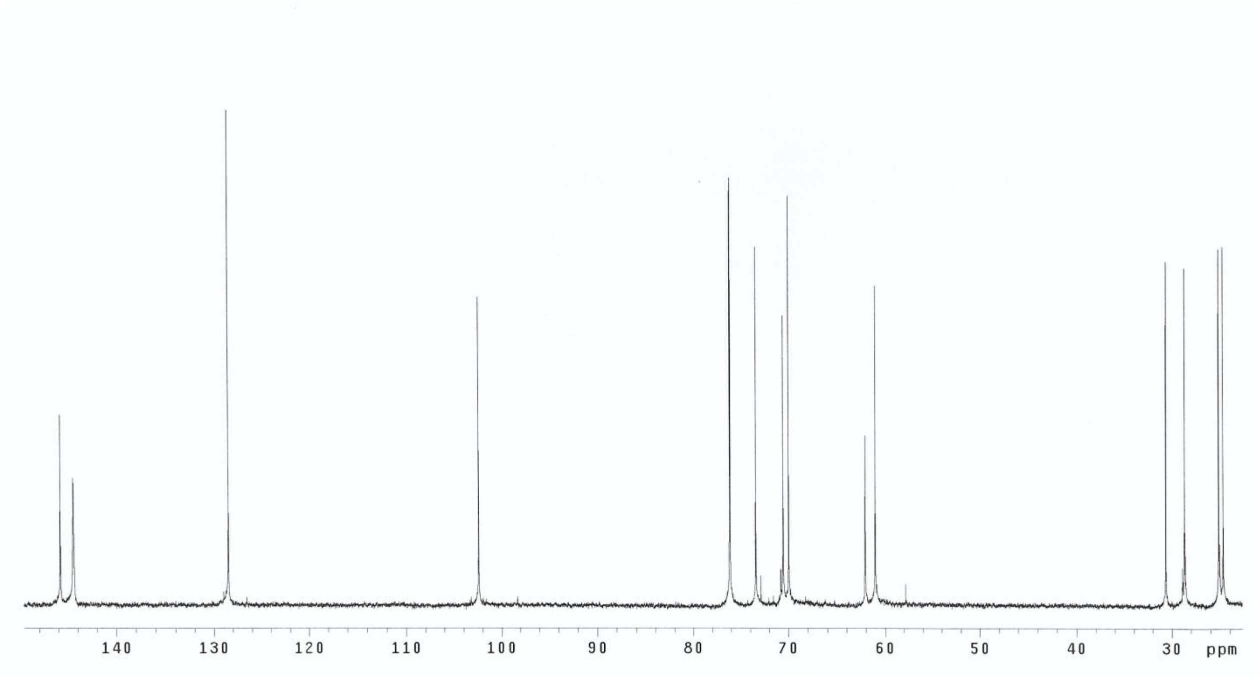

7. *N*-[6-(2',3',4',6'-Tetra-*O*-acetyl-α-D-glucopyranosyloxy)hexyl]-*N*,*N*,*N*-trimethylammonium bromide (**6a**)

^{1}H NMR (400 MHz, D_2O):

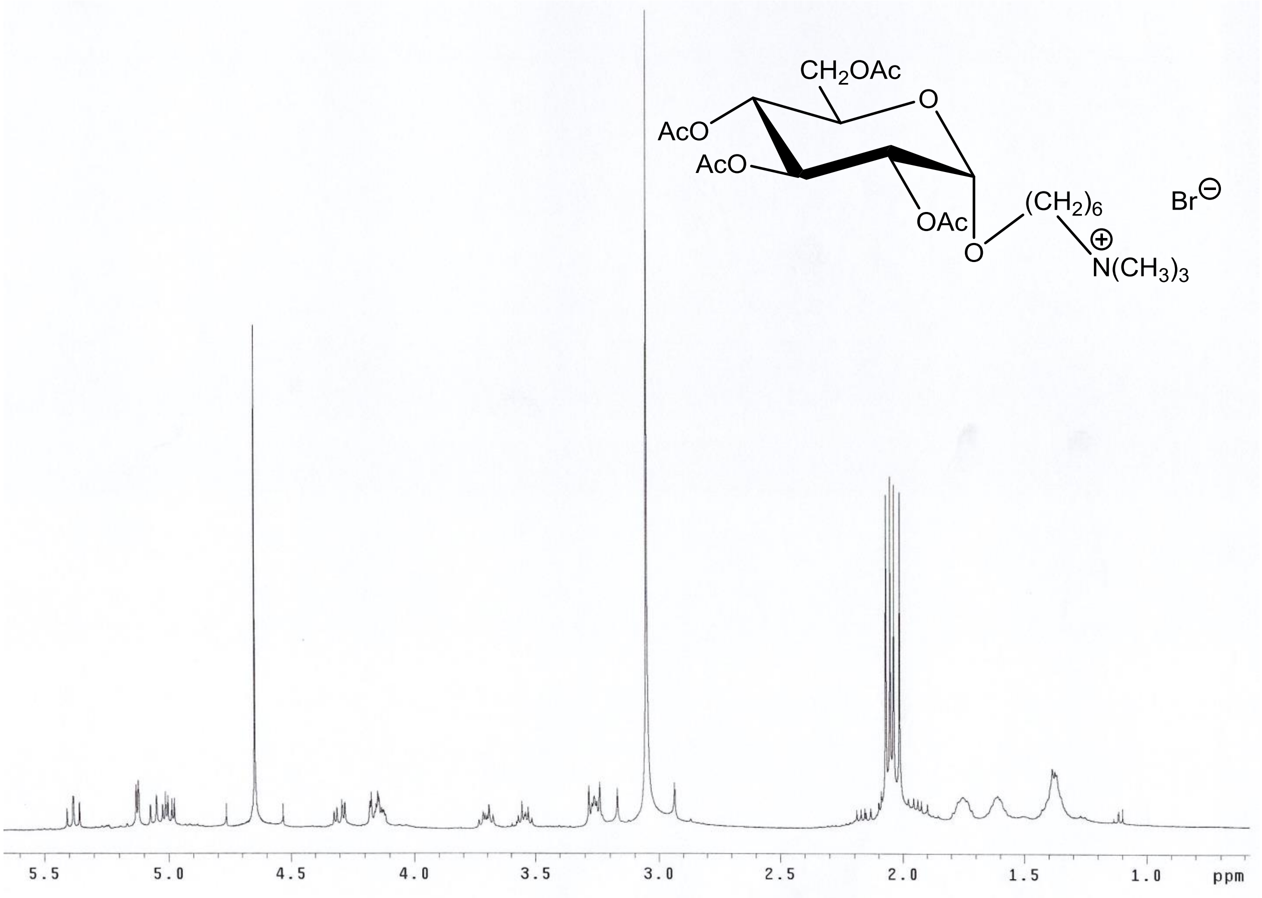

^{13}C NMR (100 MHz, D_2O):

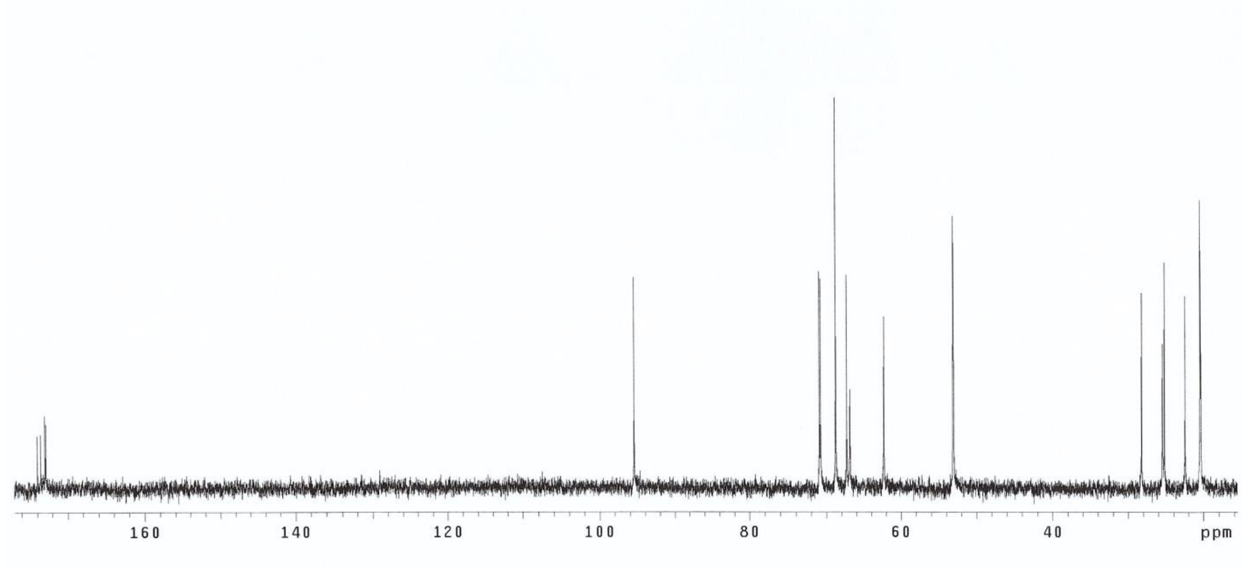

8. *N*-[6-(2',3',4',6'-Tetra-*O*-acetyl-α-D-glucopyranosyloxy)hexyl]pyridinium bromide (**6b**)

^{1}H NMR (400 MHz, D_2O):

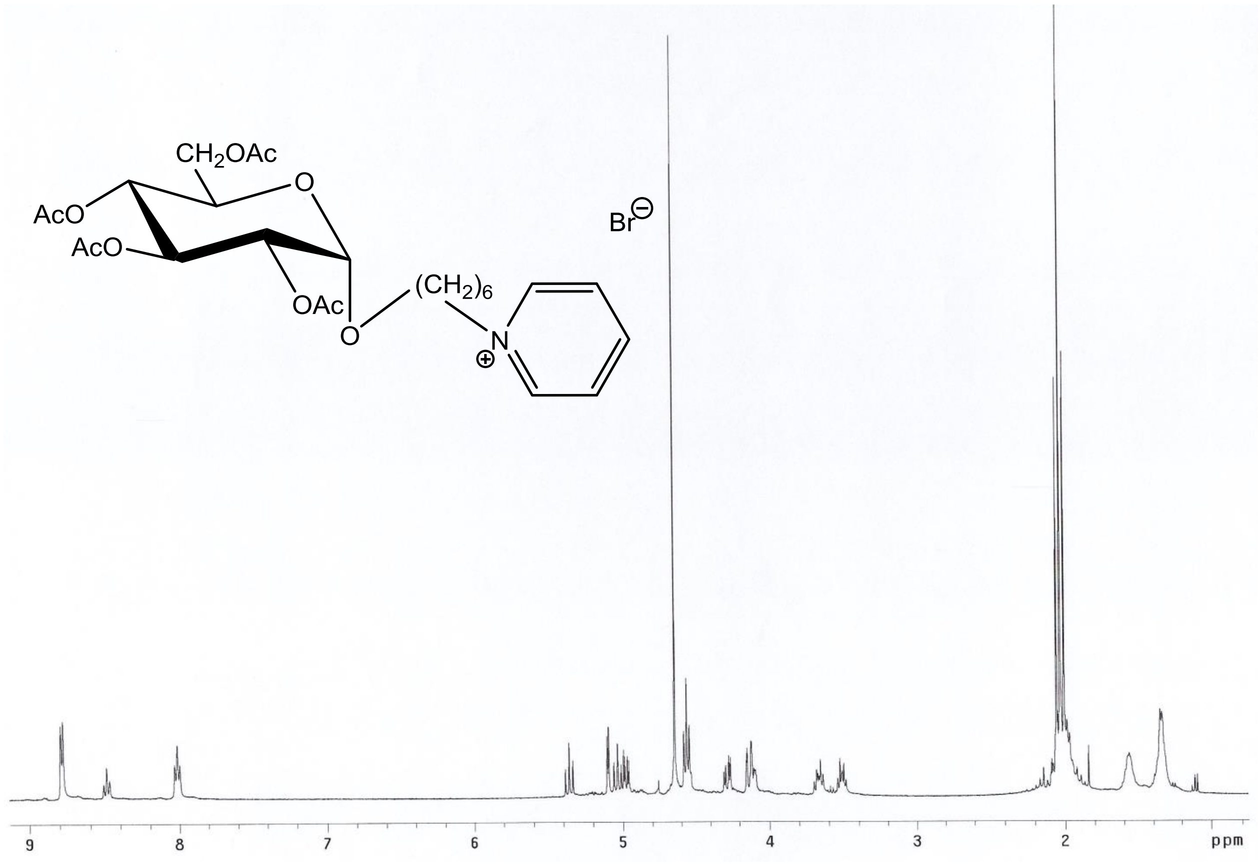

^{13}C NMR (100 MHz, D_2O):

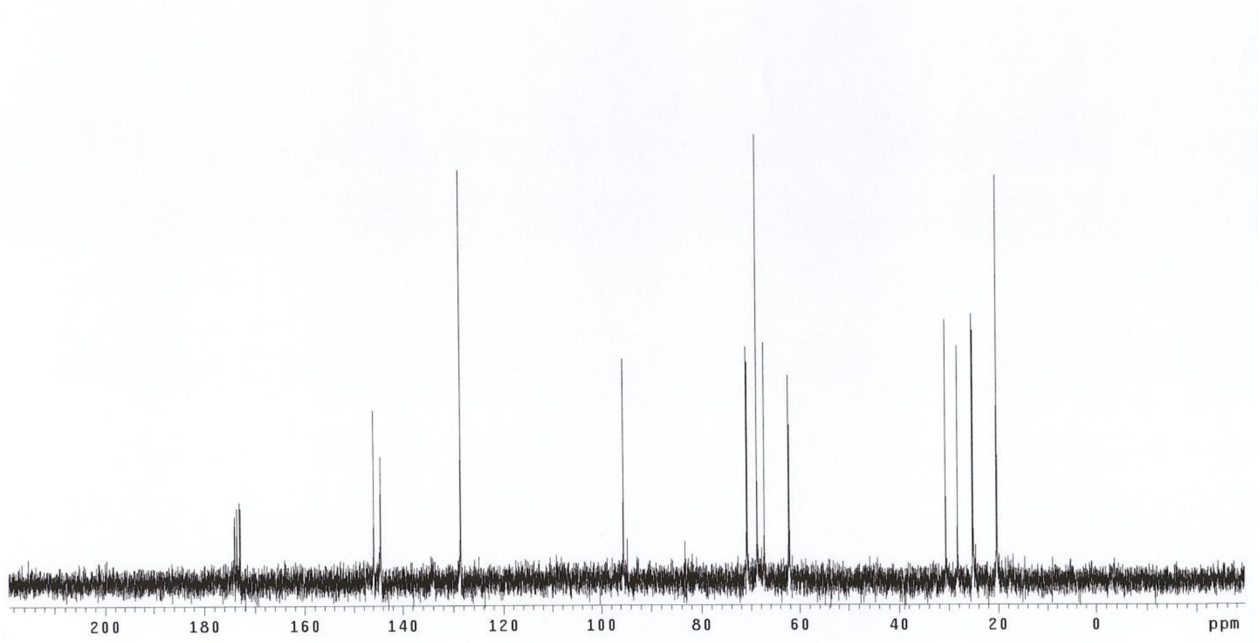

9. *N*-[6-(α-D-glucopyranosyloxy)hexyl]-*N*,*N*,*N*-trimethylammonium bromide (**7a**)

^{1}H NMR (400 MHz, D_2O):

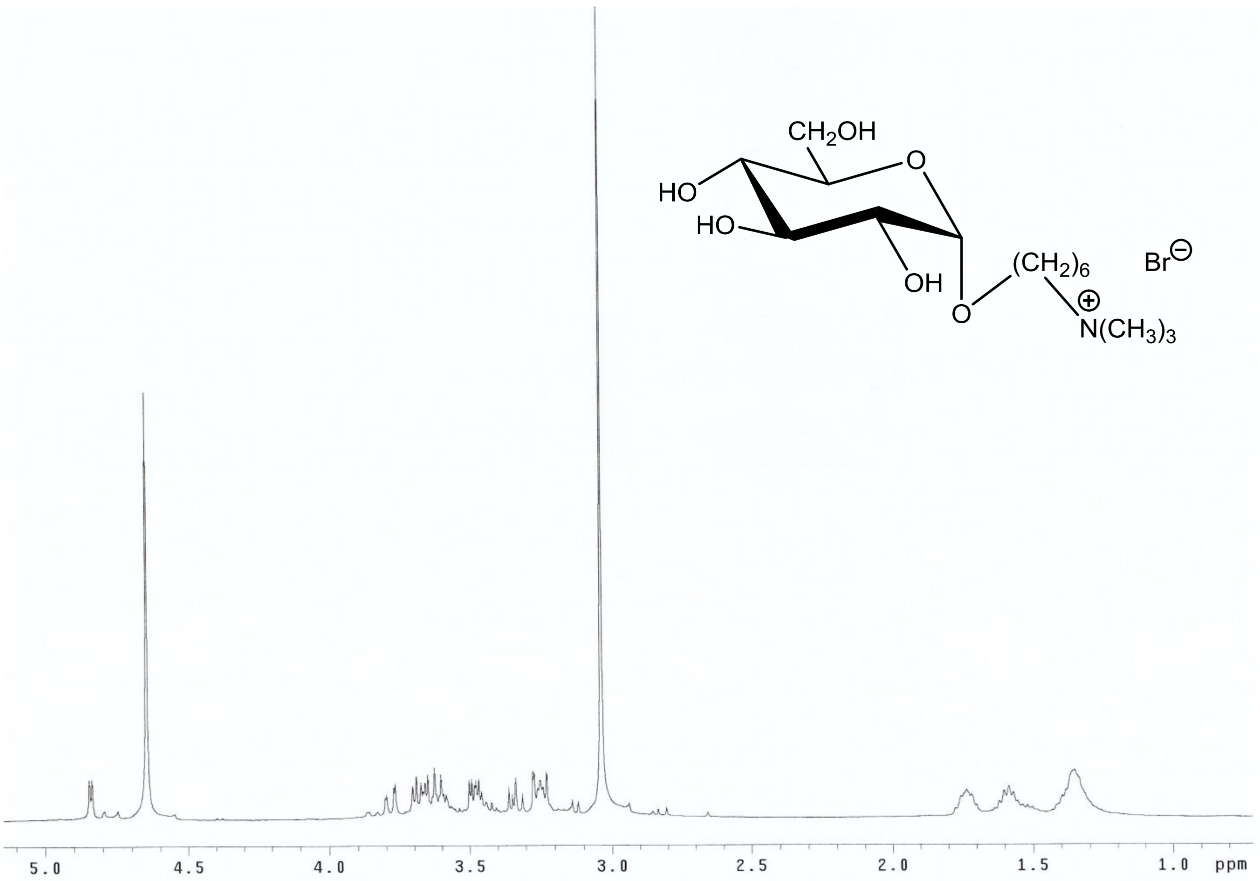

^{13}C NMR (100 MHz, D_2O):

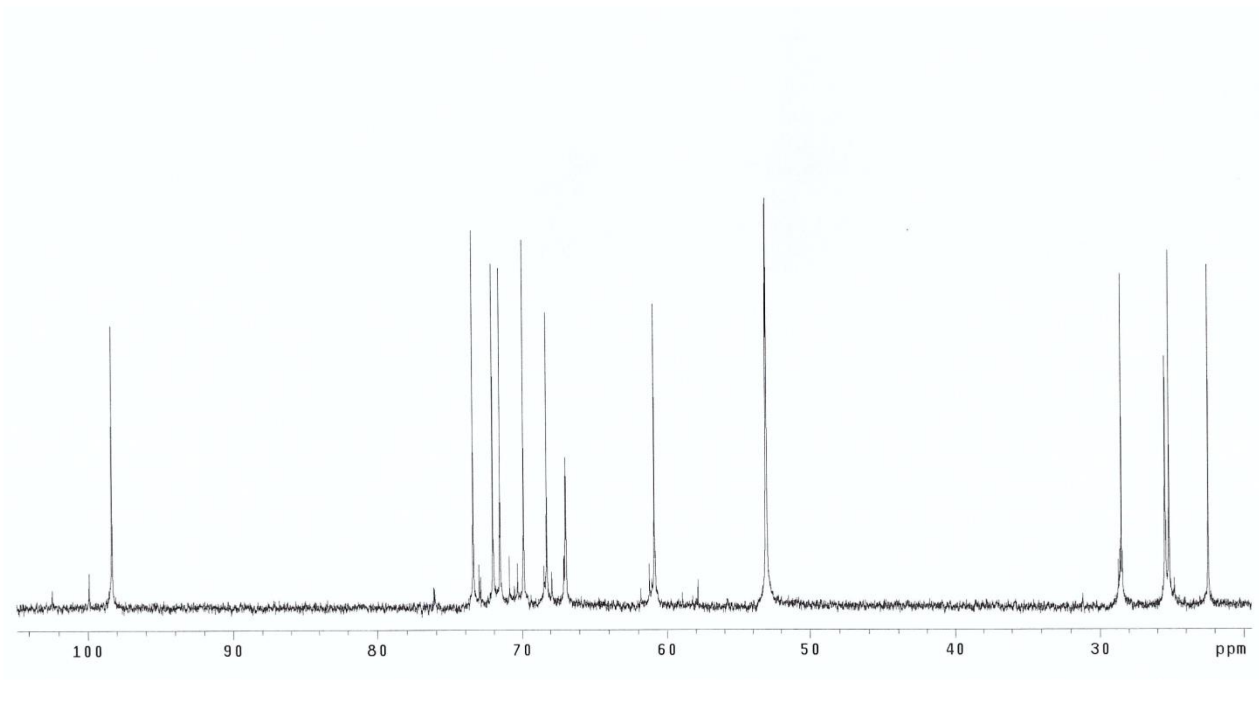

10. *N*-[6-(α-D-glucopyranosyloxy)hexyl]pyridinium bromide (**7b**)

^{1}H NMR (400 MHz, D_2O):

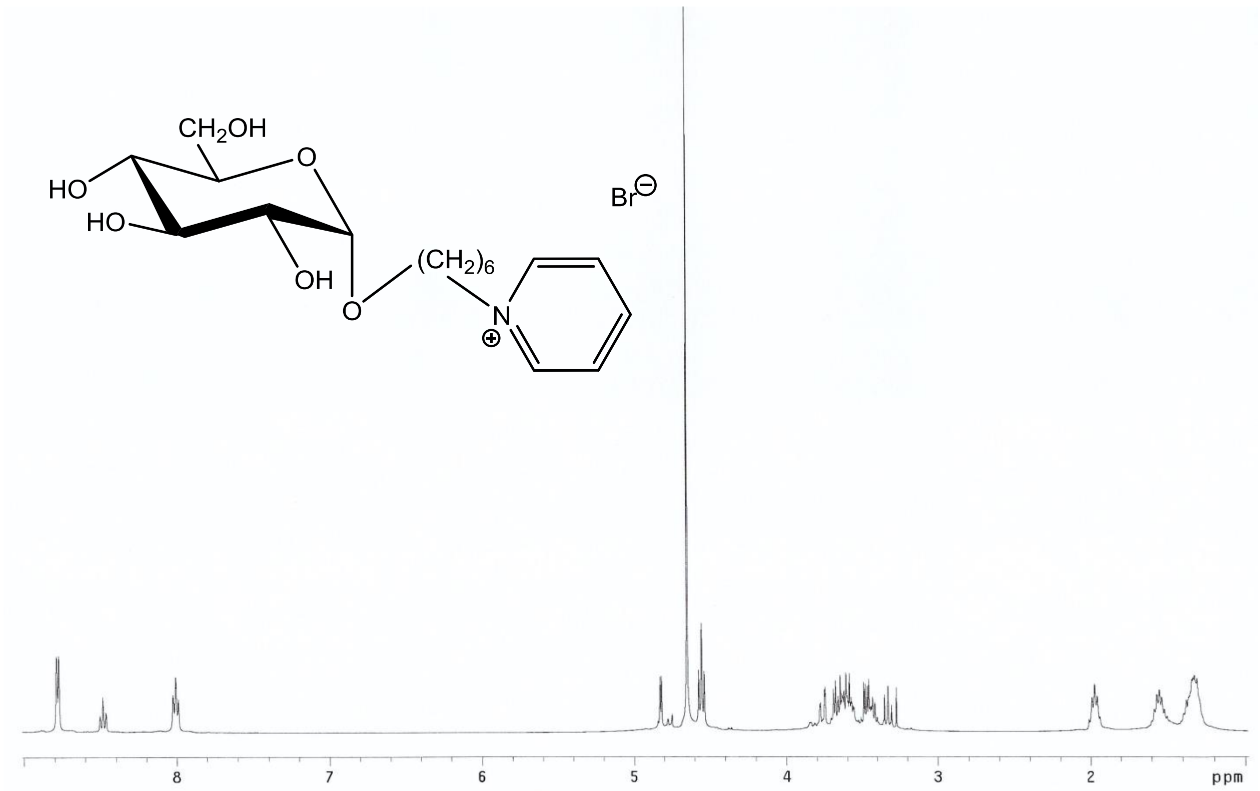

^{13}C NMR (100 MHz, D_2O):

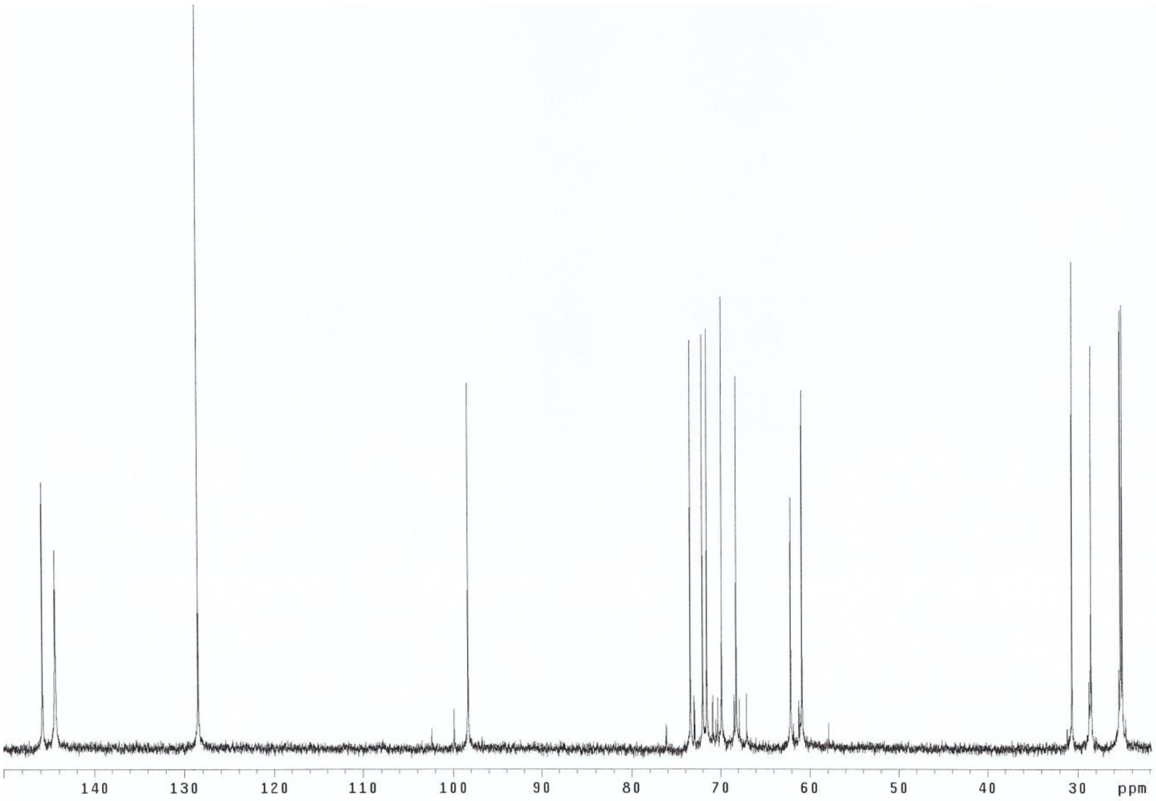

MS SPECTRA

1. *N*-[6-(2',3',4',6'-Tetra-*O*-acetyl-β-D-glucopyranosyloxy)hexyl]-*N*,*N*,*N*-trimethylammonium bromide (**4a**) m/z 490.2643 ($[M\text{-}Br]^+$)

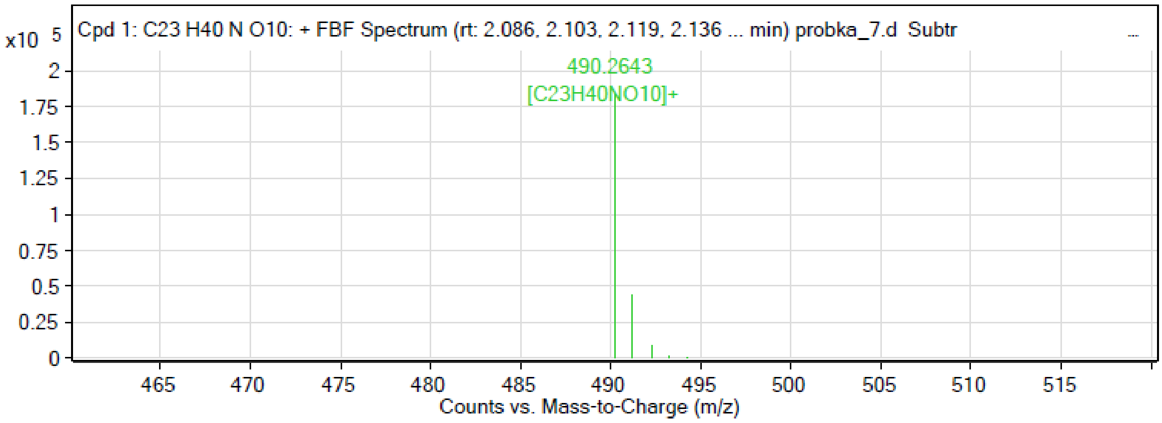

2. *N*-[6-(2′,3′,4′,6′-Tetra-*O*-acetyl-β-D-glucopyranosyloxy)hexyl]pyridinium bromide (**4b**) m/z 510.2329 ($[M\text{-}Br]^+$)

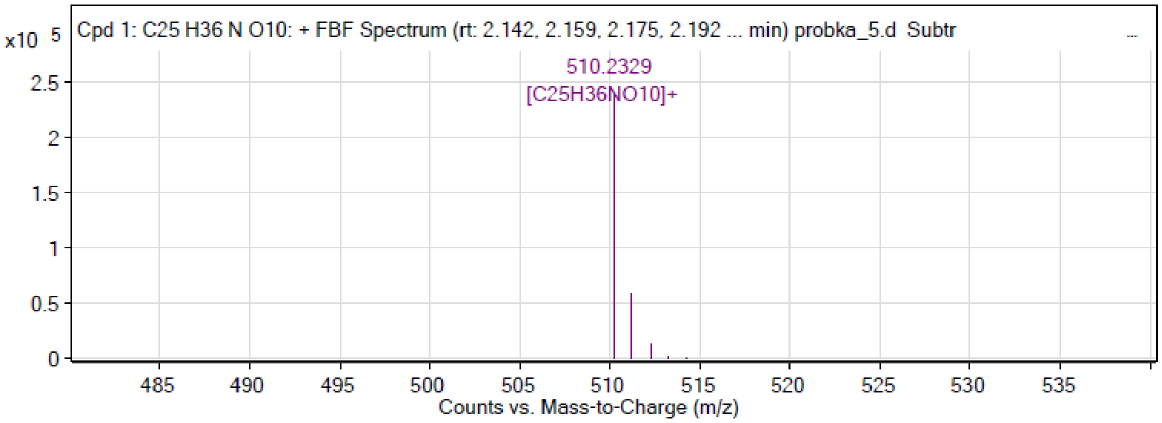

3. *N*-[6-(β-D-Glucopyranosyloxy)hexyl]-*N*,*N*,*N*-trimethylammonium bromide (**5a**) m/z 322.2223 ([M-Br]$^+$)

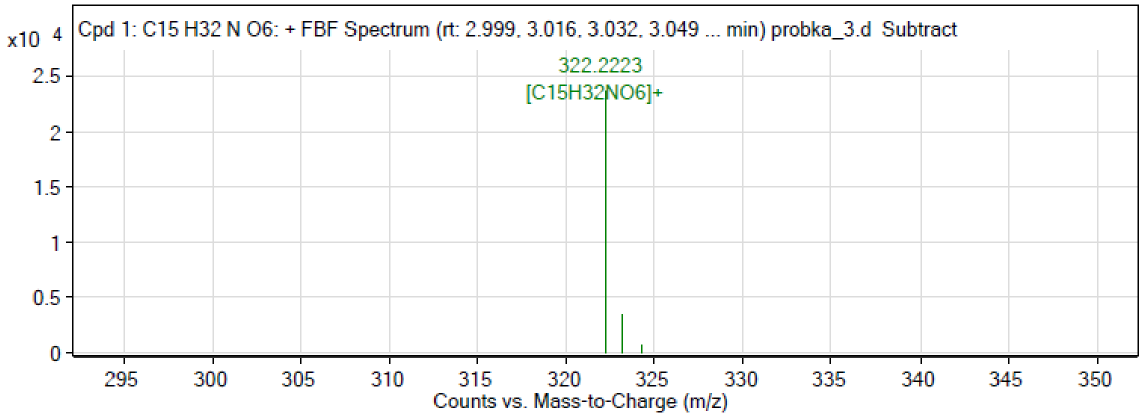

4. *N*-[6-(β-D-Glucopyranosyloxy)hexyl]pyridinium bromide (**5b**) m/z 342.1909 ([M-Br]$^+$)

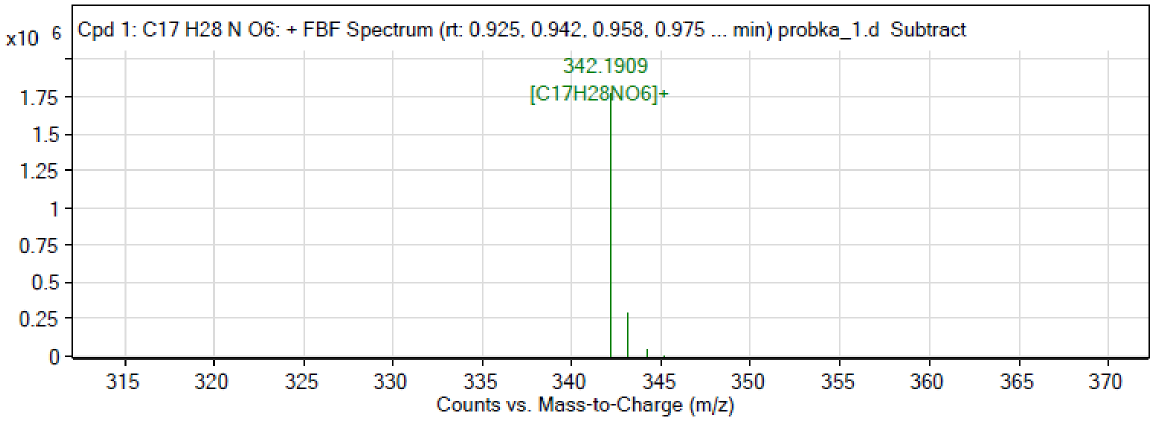

5. *N*-[6-(2',3',4',6'-Tetra-*O*-acetyl-α-D-glucopyranosyloxy)hexyl]-*N*,*N*,*N*-trimethylammonium bromide (**6a**) m/z 490.2641 ([M-Br]$^+$)

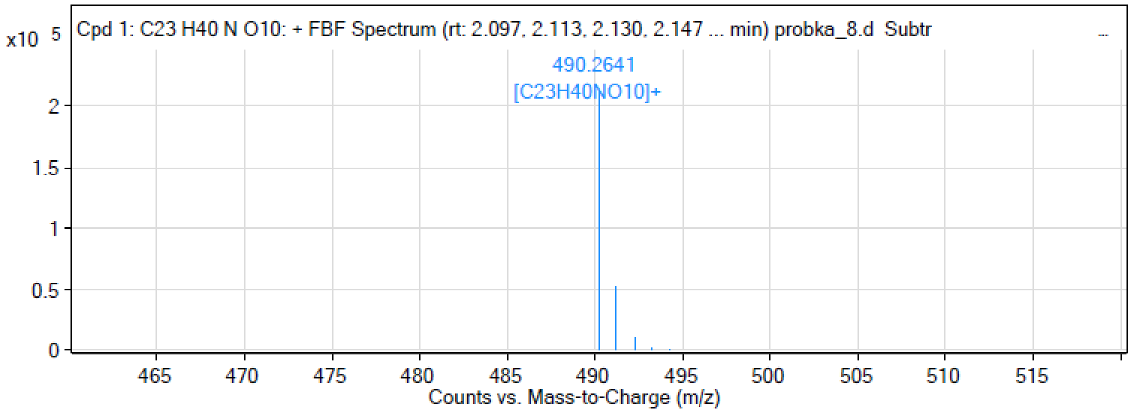

6. *N*-[6-(2',3',4',6'-Tetra-*O*-acetyl-α-D-glucopyranosyloxy)hexyl]pyridinium bromide (**6b**) m/z 510.2330 ([M-Br]$^+$)

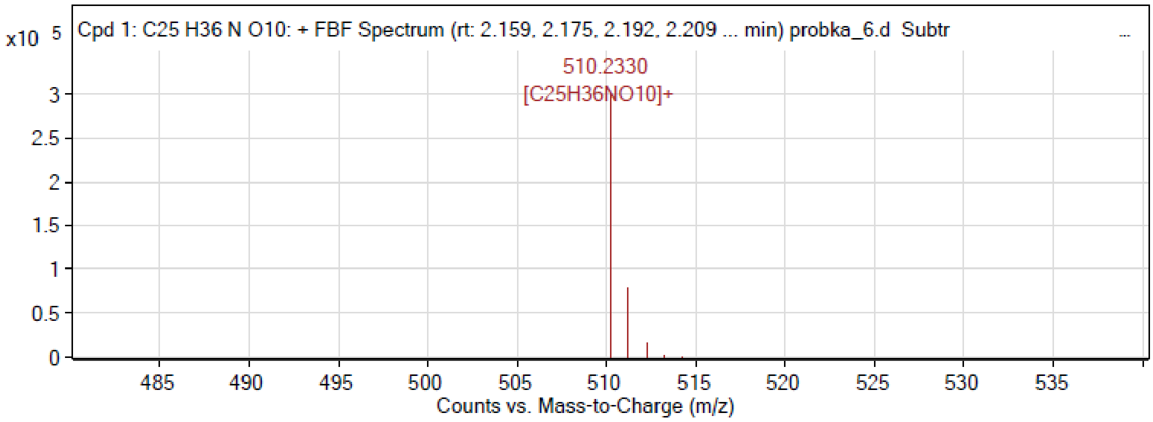

7. *N*-[6-(α-D-Glucopyranosyloxy)hexyl]-*N*,*N*,*N*-trimethylammonium bromide (**7a**) m/z 322.2222 ([M-Br]$^+$)

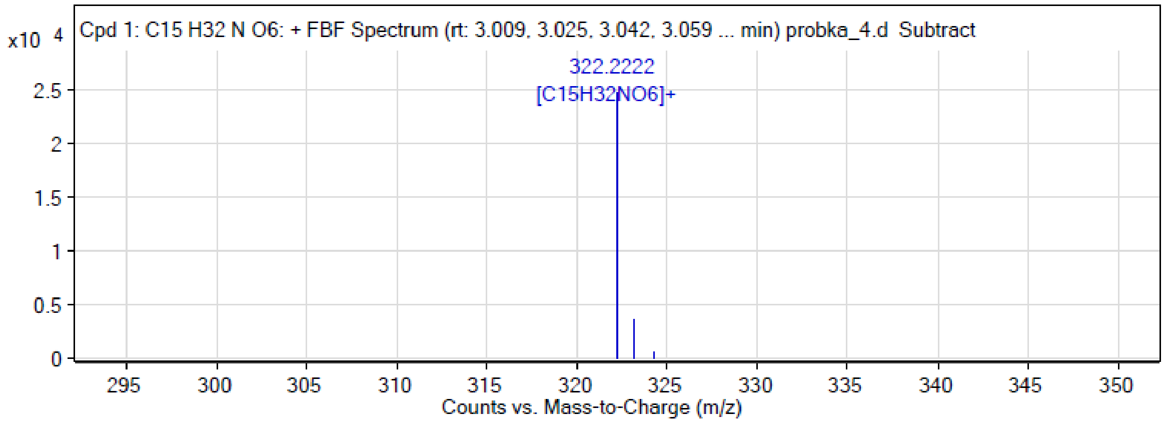

8. *N*-[6-(α-D-Glucopyranosyloxy)hexyl]pyridinium bromide (**7b**) m/z 342.1909 ([M-Br]$^+$)

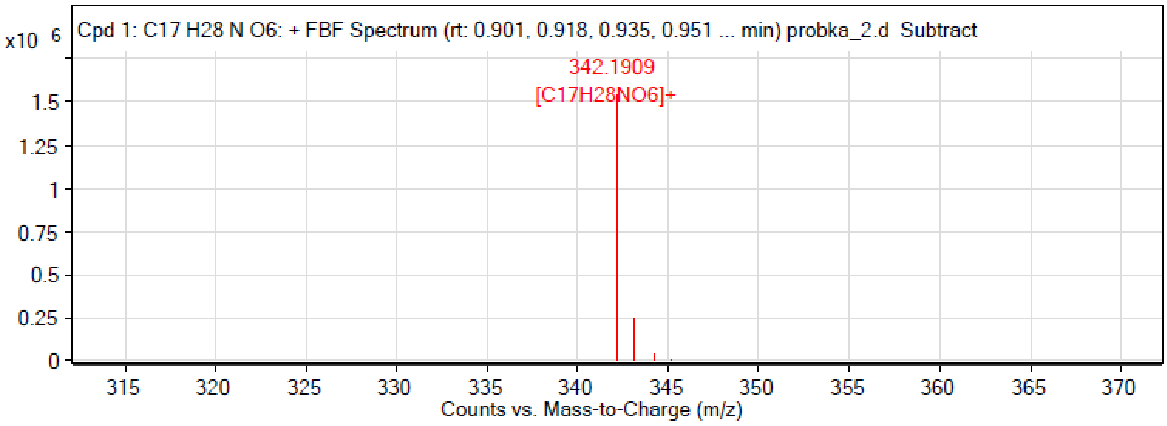

References

S1. Dubber M.; Lindhorst T. *J. Org. Chem.* **2000**, *65*, 5275-5281. doi: 10.1021/jo000432s

S2. Dmochowska B.; Piosik J.; Woziwodzka A.; Sikora K.; Wiśniewski A.; Węgrzyn G. *J. Hazard. Mater.* **2011**, *19*, 3272-278. doi:10.1016/j.jhazmat.2011.07.064

S3. Podgórska B.; Węgrzyn G. *Lett. Appl. Microbiol.* **2006**, *42*, 578-582. doi: 10.1111/j.1472-765X.2006.01891.x

S4. Woziwodzka A.; Gwizdek-Wiśniewska A.; Piosik J. *Bioorg. Chem.* **2011**, *39*, 10-17. doi: 10.1016/j.bioorg.2010.11.001

S5. Maron D.M.; Ames B.N. *Mutat.Res.* **1983**, *113*, 173-215. doi:10.1016/0165-1161(83)90010-9

S6. Mortelmans K.; Zeiger E. *Mutat.Res.* **2000**, *455*, 29-60. doi: 10.1016/S0027-5107(00)00064-6

S7. Woziwodzka A.; Gołuński G.; Wyrzykowski D.; Kaźmierkiewicz R.; Piosik J. *Chem. Res. Toxicol.* **2013**, *26*, 1660-1673. doi: 10.1021/tx4002513
